# Anthocyanin-Related Pigments: Natural Allies for Skin Health Maintenance and Protection

**DOI:** 10.3390/antiox10071038

**Published:** 2021-06-28

**Authors:** Patrícia Correia, Paula Araújo, Carolina Ribeiro, Hélder Oliveira, Ana Rita Pereira, Nuno Mateus, Victor de Freitas, Natércia F. Brás, Paula Gameiro, Patrícia Coelho, Lucinda J. Bessa, Joana Oliveira, Iva Fernandes

**Affiliations:** LAQV-REQUIMTE, Department of Chemistry and Biochemistry, Faculty of Sciences, University of Porto, 4169-007 Porto, Portugal; patricia.correia@fc.up.pt (P.C.); paula.araujo@fc.up.pt (P.A.); up201503157@fc.up.pt (C.R.); helder.oliveira@fc.up.pt (H.O.); anarita@fc.up.pt (A.R.P.); nbmateus@fc.up.pt (N.M.); vfreitas@fc.up.pt (V.d.F.); nbras@fc.up.pt (N.F.B.); agsantos@fc.up.pt (P.G.); patriciaines20@gmail.com (P.C.); lucindabessa12@gmail.com (L.J.B.)

**Keywords:** natural bioactives, anthocyanins, photoprotection, UV-filter, oxidative stress, antimicrobial, skin aging, ECM, topical formulations, cosmeceuticals

## Abstract

Human skin is commonly described as a particularly dynamic and complex environment, with a physiological balance continuously orchestrated by numerous internal and external factors. Intrinsic aging, exposure to UV radiation and skin pathogens are some of the key players that account for dermatological alterations and ailments. In this regard, this study intended to explore the potential skin-health beneficial properties of a group of molecules belonging to the anthocyanin family: cyanidin- and malvidin-3-*O*-glucosides and some of their structurally related pigments, resulting in a library of compounds with different structural properties and color hues. The inclusion of both purified compounds and crude extracts provided some insights into their distinctive effects when tested as individual agents or as part of multicomponent mixtures. Overall, most of the compounds were found to reduce biofilm production by *S. aureus* and *P. aeruginosa* reference strains, exhibit UV-filter capacity, attenuate the production of reactive oxygen species in human skin keratinocytes and fibroblasts and also showed inhibitory activity of skin-degrading enzymes, in the absence of cytotoxic effects. Carboxypyranocyanidin-3-*O*-glucoside stood out for its global performance which, combined with its greater structural stability, makes this a particular interesting compound for potential incorporation in topical formulations. Results provide strong evidence of the skin protective effects of these pigments, supporting their further application for cosmeceutical purposes.

## 1. Introduction

The importance of human skin goes way beyond its physical protective barrier role between body and surroundings, as it serves other crucial functions, including prevention of percutaneous water loss and immune surveillance [1]. Maintaining a good skin appearance is important, not only from a health standpoint but also for self-esteem and well-being since its appearance is inevitably linked to the visual perception of vitality. From a holistic perspective, skin can be deemed as a highly dynamic environment, whose overall condition is modulated by a complex network of cellular and molecular events that result from the interplay of a multitude of external (e.g., sun-light exposure and tobacco smoke) and internal factors (e.g., genetics and endocrine metabolism) [2]. The decay of its function and structural integrity naturally arises with intrinsic aging, in a sequence of events that affects both the epidermal and dermal layers of the skin, with the most prominent changes occurring in the latter, where the levels of essential components of the extracellular matrix (ECM), namely collagen and elastin, along with hyaluronic acid, gradually decline [3]. These changes are mostly driven by the continuous age-dependent accumulation of reactive oxygen species (ROS) in the skin, which activate specific cell signaling cascades that simultaneously up-regulate the expression of ECM degrading enzymes and down-regulate the synthesis of ECM constituents [4]. As a result, the levels of mechanical resistance of the ECM decrease, and so does the mechanical tension within dermal fibroblasts, disturbing their normal shape, size and function. Cells respond by increasing their intracellular levels of ROS, creating a self-perpetuating detrimental cycle, culminating in the appearance of wrinkles, skin dryness, roughness and laxity [1,5,6]. Solar ultraviolet radiation (UVR) cumulative exposure is one of the main culprits of overproduction of ROS in the skin, and also promotes inflammation, two processes that feed into one another and exacerbate the above-mentioned cell signaling pathways. Continuous UV-induced oxidative damage to cellular proteins, membranes and DNA contributes to the deep structural and functional changes inflicted on the skin, characteristic of so-called photoaging [7,8].

Cutaneous microbiota, mostly made up of bacteria, also plays a crucial role in the maintenance of dermatological health and commensal bacteria are important in the mediation of skin physiological processes. However, in situations where the integrity of the epidermal barrier is compromised or in dysbiotic conditions, the skin becomes susceptible to bacterial colonization, infection and the possible onset of serious and difficult to irradicate skin disorders [9].

Given the increasing awareness of the importance of skin health, there is a growing demand for efficient solutions for preventing and treating skin related damage and disorders. Within this context, the use of compounds derived from natural sources for dermatological applications has gone through an exponential growth in popularity [10]. There seems to be a clear trend shift in consumer preference for these types of natural ingredients, which has boosted the research for innovative combinations of these bioactives. Amongst the existing classes of phytochemicals, anthocyanins represent one of the most attractive, owing to their simultaneous acknowledged bioactivity repertoire and visually attractive colors [11]. The growing and compelling evidence of the biological relevance of these polyphenolic compounds for skin-related applications has raised the interest in their use as cosmeceuticals [12,13,14,15].

**Figure 1:**
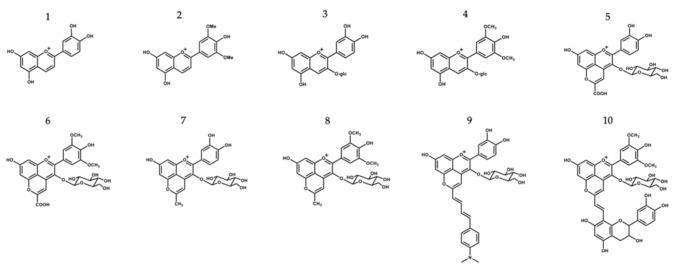
Chemical structure compounds (deoxyanthocyanins, anthocyanins and its related structures) selected for this study: 1—Luteolinidin (Lut); 2—Deoxymalvidin (DeoxyMv); 3—Cyanidin-3-*O*-glucoside (Cy-3-glc); 4—Malvidin-3-*O*-glucoside (Mv-3-glc); 5—Carboxypyranocyanidin-3-*O*-glucoside (CarboxypyCy-3-glc); 6—Carboxypyranomalvidin-3-*O*-glucoside (CarboxypyMv-3-glc); 7—Methylpyranocyanidin-3-*O*-glucoside (MethylpyCy-3-glc); 8—Methylpyranomalvidin-3-*O*-glucoside (MethylpyMv-3-glc); 9—4-(Dimethylamino)-cinnamyl-10-butadienylidene-pyranocyanidin-3-*O*-glucoside (Dimethylamino-cin-but-pyCy-3-glc); 10—Vinylpyranomalvidin-3-*O*-glucoside-catechin (VinylpyMv-3-glc-catechin).

The purpose of this study was to assess the potential protective and health promoting skin effects of a group of molecules belonging to the same anthocyanin family, including their antimicrobial activity, UV-filter capacity and inhibitory action on ROS production and skin-degrading enzymes. Cyanidin- and malvidin-3-*O*-glucosides and their corresponding deoxyanthocyanins (which lack the substitution at the C-3 position, making these pigments much less sensitive to water addition at C2) were included in this study, along with some pyranoanthocyanins structures (commonly found in wine matrices during the aging process and known for their higher structural and color stability in a wider pH range), which were obtained by further chemical modifications of the two native anthocyanins, giving rise to a library of compounds with distinct structural and chromatic properties [16,17] (Figure 1). Purified compounds were used to understand the full potential of each and to establish some structure–activity relationships, while extracts were also included in some of the analyses to investigate possible existing synergy or antagonism effects in complex multicomponent mixtures.

## 2. Materials and Methods

### 2.1. Isolation and Synthesis of the Different Compounds

Deoxyanthocyanins were synthesized by acidic aldol condensation and consisted of the mixture of 2,4,6–trihydroxybenzaldehyde with 3,4-dihydroxyacetophenone and 3′,5′-dimethoxy-4′-hydroxyacetophenone (for luteolinidin and deoxymalvidin synthesis, respectively) [18]. Cyanidin- and malvidin-3-*O*-glucosides were obtained by the fractionation of blackberries and young red wine extract, and their further reaction with pyruvic acid (molar ratio of 1:100) and acetone (10% *v/v* aqueous solution) resulted in the formation of the carboxy and methylpyrano extracts, respectively, as described previously [19,20]. The obtained carboxypyranoanthocyanin extracts were tested as a complex mixture and also as a means of purifying carboxypyranocyanidin-3-*O*-glucoside and carboxypyranomalvidin-3-*O*-glucoside. The characterization of the extracts in terms of protein, lipids, sugar and total phenolic content and antioxidant activity is available in Supplementary Material (Table S1).

Amino-derived pyranocyanidin-3-*O*-glucoside was obtained from the reaction of methylpyranocy-3-glc with 4-(dimethylamino)-cinnamaldehyde as reported elsewhere [21]. The formation of vinylpyranomalvidin-3-*O*-glucoside-catechin resulted from the mixture of the carboxypyranomalvidin-3-*O*-glc with (+)-catechin, in the presence of acetaldehyde [22].

Synthesis reactions and the purity of the compounds were monitored by HPLC-DAD.

### 2.2. Antimicrobial and Antibiofilm Assays

#### 2.2.1. Bacterial Strains and Growth Conditions

Compounds/extracts were tested against the following bacterial strains: *Pseudomonas aeruginosa* ATCC 27853, *Staphylococcus aureus* ATCC 29213, *Staphylococcus epidermidis* ATCC 14990, *Streptococcus pyogenes* ATCC 19615, and *Micrococcus luteus* ATCC 4698. Prior to each in vitro bioassay, fresh cultures were obtained for each strain using the appropriate medium and incubation conditions as follows. *Staphylococcus* spp. and *P. aeruginosa* were grown on Mueller–Hinton (MH) agar (Liofilchem srl, Roseto degli Abruzzi, Italy) for 24 h at 37 °C, while *M. luteus* was grown in Tryptic Soy agar (TSA, Liofilchem srl, Italy) for 24 h at 30 °C and *S. pyogenes* on TSA supplemented with 5% defibrinated sheep blood (Thermo Fisher Scientific, Waltham, MA, USA) for 24 h at 37 °C in an atmosphere of 5% CO_2_.

#### 2.2.2. Determination of Minimum Inhibitory and Minimum Bactericidal Concentrations

Minimum inhibitory concentrations (MICs) of compounds/extracts were determined using a broth microdilution technique, following the recommendations of the Clinical and Laboratory Standards Institute [23]. Briefly, fresh colonies of each strain were used to prepare the respective inocula with an optical density of 600 nm (OD_600_) equal to 0.1 (approximately 1 × 10^8^ CFU/mL). For all strains, cation-adjusted Mueller–Hinton broth (MHB2, Sigma-Aldrich, St. Louis, MO, USA) was used, but in the case of *S. pyogenes*, MBH2 was previously supplemented with 2.5% lysed horse blood (Thermo Fisher Scientific, USA). In 96-well, U-bottom microplates, each compound was serially diluted in the respective medium from stock solutions (10 mg/mL in DMSO) to achieve in-test concentrations ranging from 0.5 to 512 μg/mL. Wells were inoculated so that the final concentration in each was 5 × 10^5^ CFU/mL. The plates were then incubated at 37 °C for 24 h. MIC was defined as the lowest concentration of compound inhibiting the bacterial growth visible to the naked eye. The concentration of DMSO in the highest in-test concentration did not affect the bacterial growth of the tested strains. Minimum bactericidal concentration (MBC) was determined by spreading 10 μL on MH agar (or blood agar in the case of *S. pyogenes*) from each well showing no visible growth, with further incubation for 24 h at 37 °C; the lowest concentration at which no growth occurred on MH plates was defined as the MBC.

#### 2.2.3. Biofilm Formation Inhibition Assay

The capacity of all compounds/extracts to interfere with the biofilm formation by *P. aeruginosa* ATCC 27853 and *S. aureus* ATCC 29213 was assessed, using the crystal violet assay as previously reported [24]. TSB was used for *P. aeruginosa* ATCC 27853, whereas TSB supplemented with 1% glucose (TSBG) was used for *S. aureus* ATCC 29213. All compounds and extracts were used at three concentrations (256, 64 and 16 µg/mL), which were necessarily sub-inhibitory concentrations (below the MIC).

### 2.3. Determination of Solar Protection Factor (SPF)

To determine the in vitro solar protection factor (SPF), a relative measure of UVB protection, 1.0 mg of each compound/extract was weighed and diluted in ethanol (0.2 mg/mL), followed by ultra-sonication for 5 min. The absorption spectrum of each sample was collected in the range of 290 to 320 nm (every 5 nm), with 3 measurements for each point, using a 1 mm quartz cell and ethanol as blank. Additionally, SPF values were also determined in the presence of a conventional chemical UV filter, oxybenzone (Sigma-Aldrich, Spain), at a concentration of 0.1 mg/mL. Values were calculated according to Mansur equation:

$$SPF_{spectrophotometric}=CF\times \overset{320}{\underset{290}{\sum }}EE\;\left(\lambda \right)\times I\left(\lambda \right)\times Abs\;\left(\lambda \right)$$

where the *CF* (correction factor) is 10, *EE (λ)* represents the erythmogenic effect of radiation with wavelength *λ*, and *Abs* refers to spectrophotometric absorbance values at each wavelength *λ*. *EE(λ)* × *I(λ)* values are constant and are presented in Table 1.

### 2.4. Cell Culture Assays

#### 2.4.1. Cell Lines and Growth Conditions

Primary Normal Human Epidermal Keratinocytes (HeKa) (ATCC^®^ PCS200011™) were grown in Dermal Cell Basal Media (ATCC^®^ PCS200030) supplemented with Keratinocyte Growth Kit components (ATCC^®^ PCS200040) and 0.1% antibiotic/antimycotic solution (10 units/mL of penicillin, 10 μg/mL of streptomycin and 25 ng/mL of amphotericin B, from Sigma-Aldrich) at 37 °C in a humidified atmosphere with 5% CO_2_. Cells were harvested using Trypsin-EDTA for Primary Cells (ATCC PCS999003) once a week, at 80% confluence.

Spontaneously transformed aneuploid immortal keratinocyte cell lines from adult human skin (HaCat) and human foreskin fibroblasts (HFF-1) were cultured in 22.1 cm^2^ plates and maintained in Dulbecco’s Modified Eagle Medium (DMEM, Cell Lines Service), supplemented with 10% or 15% fetal bovine serum (FBS, CLS) and 1% of antibiotic/antimycotic solution (100 units/mL of penicillin, 10 mg/mL of streptomycin and 0.25 mg/mL of amphotericin B, Sigma-Aldrich, St. Louis, MO, USA), at 37 °C in an atmosphere of 5% CO_2_. Medium was renewed every two days and cells were harvested every two weeks.

#### 2.4.2. Cytotoxicity Evaluation

Possible cytotoxic effects of the compounds towards HaCat and HFF-1 cells were evaluated using the standard MTT assay. Briefly, cells were seeded at a density of 5 × 10^4^ (HaCat) and 1.4 × 10^4^ cells/mL (HFF-1) onto 96-well plate and incubated for 24 h to allow cell attachment. Then, serially diluted compound solutions (0.78–100 µM) were added to the wells. Following a period of incubation of 48 h at 37 °C, wells were washed once with phosphate buffered saline (PBS, Sigma-Aldrich) and MTT solution (0.45 mg/mL) was added to each well. After 1.5 h of incubation, medium was discarded, and the formed formazan crystals were dissolved with dimethylsulfoxide (DMSO, Sigma-Aldrich). Absorbance was read at 570 nm (Biotek PowerWave XS, Winooski, VT, USA).

#### 2.4.3. ROS Formation Assay

Reactive oxygen species production in HFF-1 and HaCat cells was evaluated following the standard method. Cells were seeded at a density of 5 × 10^4^ cells/well onto 96-well plates and allowed to reach confluency. At this point, cells were washed twice with phosphate buffered saline solution (PBS, Sigma-Aldrich, St. Louis, MO, United States) and compounds were added at a concentration of 50 µM, followed by 24 h of incubation at 37 °C in a 5% CO_2_ atmosphere. After that, cells were washed twice in Hank’s Buffered Saline Solution (HBSS) and incubated with 100 µL of 50 µM DCFDA for 30 min in the same previous conditions. Afterwards, cells were washed twice with HBSS and incubated with fresh medium for 24 h, and the fluorescence intensity (498 nm excitation/522 nm emission) was then registered on a FlexStation 3 Multi-Mode Microplate Reader (Molecular Devices).

#### 2.4.4. Transport Assay

HeKa cells were plated at a cell density of 2.6 × 10^5^ cells/mL on 12-well transwell inserts with a 0.4 μm pore size (Corning Costar, Corning, NY, USA). The culture medium was changed every two days and cells were allowed to grow and differentiate to confluent monolayers for 8 days after the initial seeding. Cells were kept for 4 days in low calcium medium (0.06 mM) to allow them to form a monolayer. For differentiation, in the last 4 days medium was changed to high calcium (1.8 mM). Only HeKa monolayers with an integrity equivalent to a transepithelial electrical resistance (TEER) higher than 450 Ω.cm^2^ were used for the transport studies. Compound solutions at different concentrations were added to the apical compartment of the cell monolayers. The chamber was incubated at 37 °C for 1, 2, 3 or 24 h, after which both apical and basolateral aliquots were taken for HPLC-MS analysis. Transport rates were calculated as follows:

$$Transport\;rate\;\%=\frac{C_{BL}}{C_{AP}}\times 100$$

where *C_BL_* represents the compound concentration at the basolateral side at a given time and *C_AP_* represents the initial compound concentration at the apical side.

### 2.5. Enzymatic Inhibition Assays

#### 2.5.1. Collagenase

A stock solution of *Clostridium histolyticum* collagenase (C0130, Sigma-Aldrich) was prepared in 100 mM of Phosphate Buffer pH 7.4 at a concentration of a 125 U/mL. Substrate N-[3-(2-furyl)-acryloyl]-Leu-Gly-Pro-Ala (FALGPA) and the different compounds were prepared in the same buffer at 6 mM and 200 μM, respectively. Compounds (75 μL) were incubated with the substrate (30 μL) for 10 min, followed by enzyme addition (40 μL) to start the reaction, which was followed by 35 min at 324 nm and 37 °C on a FlexStation 3 Multi-Mode Microplate Reader (Molecular Devices). Inhibition percentage was calculated using the following formula:

$$\mathrm{Collagenase}\;\mathrm{inhibition}\;\%=(1\;–\frac{A-B}{C-D})\times 100$$

where *A* and *B* represent the initial and final optical densities of the reaction in the presence of compounds, while *C* and *D* represent the initial and final optical densities of the reaction in the absence of compounds, respectively. Epigallocatechin-gallate (EGCG) was used as positive control at 50 μM.

#### 2.5.2. Elastase

Experiments were conducted according to Sigma-Aldrich guidelines, with minor modifications. Briefly, 75 µL of each compound solution (200 µM), 30 µL of a freshly prepared working solution of porcine pancreatic elastase (0.3 U/mL) (Sigma-Aldrich, E1250), and 175 µL of Tris-HCl buffer pH 6.80 at 25 °C were mixed in a 96-well plate and incubated for 15 min. A reaction was initiated with the addition of 20 µL of N-Succinyl-Ala-Ala-Ala-p-nitroanilide (Bachem) (4.4 mM) and the release of p-nitroanilide (p-NA) was monitored spectrophotometrically at 405 nm for 40 min at 25 °C on a FlexStation 3 Multi-Mode Microplate Reader (Molecular Devices). The inhibition rate was calculated as follows:

$$\mathrm{Elastase}\;\mathrm{inhibition}\;\%=(1\;–\frac{B-A}{D-C})\times 100$$

where *A* and *B* represent the initial and final optical densities of the reaction in the presence of compounds, while *C* and *D* represent the initial and final optical densities of the reaction in the absence of compounds, respectively. *N*-Methoxysuccinyl-Ala-Ala-Ala-Val-chloromethyl ketone (MAAPVCK) (M0398, Sigma-Aldrich) was used as a positive control at 10 μM.

#### 2.5.3. Hyaluronidase

30 µL from a stock solution of type I-S hyaluronidase from bovine testes (20 U/mL in 0.02 M phosphate buffer, pH 7, containing 50 mM NaCl) (H3506, Sigma-Aldrich) were mixed with 37.5 µL of a stock solution of each compound (200 µM) and then pre incubated for 10 min at 37 °C. Hyaluronic acid sodium salt from rooster comb (H5388, Sigma-Aldrich) was dissolved in sodium phosphate aqueous solution (0.03% in 300 mM sodium phosphate, pH 5.35) and heated at 60–70 °C for 20 min to ensure complete dissolution. After that, 30 µL of hyaluronic acid were added to the mixture and the 96-well plate was incubated at 37 °C for 60 min. Residual undigested hyaluronic acid was precipitated with 150 µL of ‘stop reaction’ solution (2.5% *w/v* CTAB in 2% *w/v* NaOH). The plate was left at room temperature for 10 min and the absorbance was measured at 400 nm on a FlexStation 3 Multi-Mode Microplate Reader (Molecular Devices, San Jose, California, USA). The percentage of inhibition was calculated as follows:

$$\mathrm{Hyaluronidase}\;\mathrm{inhibition}\;\%=(1\;–\frac{A}{B})\times 100$$

where *A* and *B* represent the enzymatic activity in the presence and absence of the compound, respectively. Quercetin at 50 µM was used as the positive control.

### 2.6. Molecular Docking

The X-ray structure of hyaluronidase of bee venom was obtained from Protein Data Bank (PDBID 1FCV at 2.65 Å resolution) [25]. This structure was chosen because it has an active site similar to the human enzyme and it has been used successfully in similar studies [25,26]. In this study, the binding of the pseudobase carbinol and chalcone forms of cyanidin-3-*O*-glucoside (Cy-3-glc), luteolinidin (Lut), malvidin-3-*O*-glucoside (Mv-3-glc) and deoxymalvidin (DeoxyMv), as well as the carboxypyranocyanidin-3-*O*-glucoside (CarboxypyCy-3-glc), methylpyranocyanidin-3-*O*-glucoside (MethylpyCy-3-glc), carboxypyranomalvidin-3-*O*-glucoside (CarboxypyMv-3-glc) and methylpyranomalvidin-3-*O*-glucoside (MethylpyMv-3-glc) were assessed. Quercetin (Que) and quercetin-3-glucoside (Que-3-glc) were used as positive controls. Ligand structures were built using the Avogadro software [27] and the molecular docking procedure was performed using the Autodock 4.2 software [28]. The docking process was validated by the re-docking of the crystallographic tetrasaccharide, consisting of two units of glucuronic acid and N-acetylglucosamine, and then the same molecular docking protocol was applied to the ligands under study. Crystallographic water molecules were removed and the hydrogen atoms were added to the enzyme, considering the protonation state of all protein residues in their physiological state (the exception was the protonated Glu219 residue). The PROPKA program [29] was used to check the p*K*_a_ values of all ionizable residues. Two disulfide bridges (Cys22-Cys313 and Cys189-Cys201) were also defined. The grid center was: X—2.606; Y—31.694; Z—−8.535 and comprised 60 × 60 × 60 points with a spacing of 0.375. The Lamarckian genetic algorithm with a total of 50 solutions was used. The population size was 150, the maximum number of evaluations was 250,000 and the number of generations was 27,000. All solutions were ranked by the ΔG_binding_ values. The best docking solutions for each ligand were analyzed using the Visual Molecular Dynamics (VMD) program [30].

### 2.7. Statistical Analysis

Results were expressed as the mean ± standard error mean (SEM) of at least 3 independent experiments. Statistical analysis was performed with GraphPad Prism 8.2.1. software, using one-way analysis of variance (one-way ANOVA) with Turkey’s test to estimate significant differences between the means of different experimental groups. Significance was set at *p* < 0.05.

## 3. Results and Discussion

### 3.1. Antimicrobial and Antibiofilm Activities

#### 3.1.1. Antibacterial Activity

Overall, the compounds and extracts tested did not show antibacterial activity (Table 2). MIC values could only be determined for Lut and dimethylamino-cin-but-pyCy-3-glc against *M. luteus* and for Lut against *S. epidermidis*, but the values presented were relatively high.

#### 3.1.2. Inhibition of Biofilm Formation

**Figure 2:**
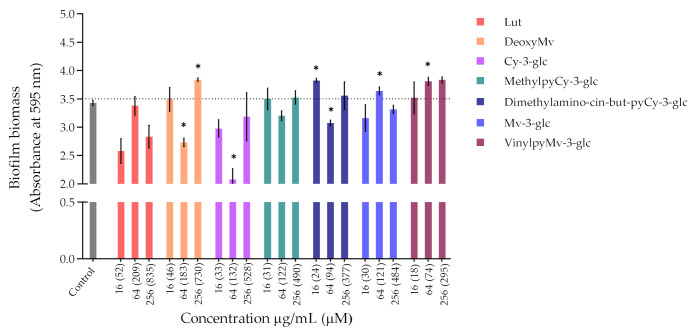

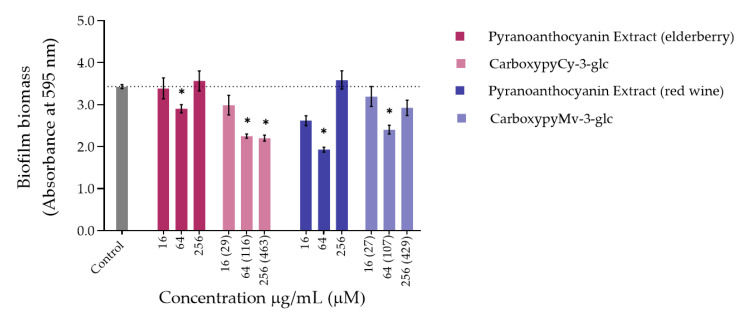
Biomass quantification of biofilms of *P. aeruginosa* ATCC 27853 formed in the absence (control) and in the presence of different concentrations of extracts or compounds: 256 µg/mL, 64 µg/mL and 16 µg/mL. Statistically significant differences between biofilms formed in presence of the extract/compound and control biofilm (*p* < 0.05) are marked with an asterisk.

**Figure 3:**
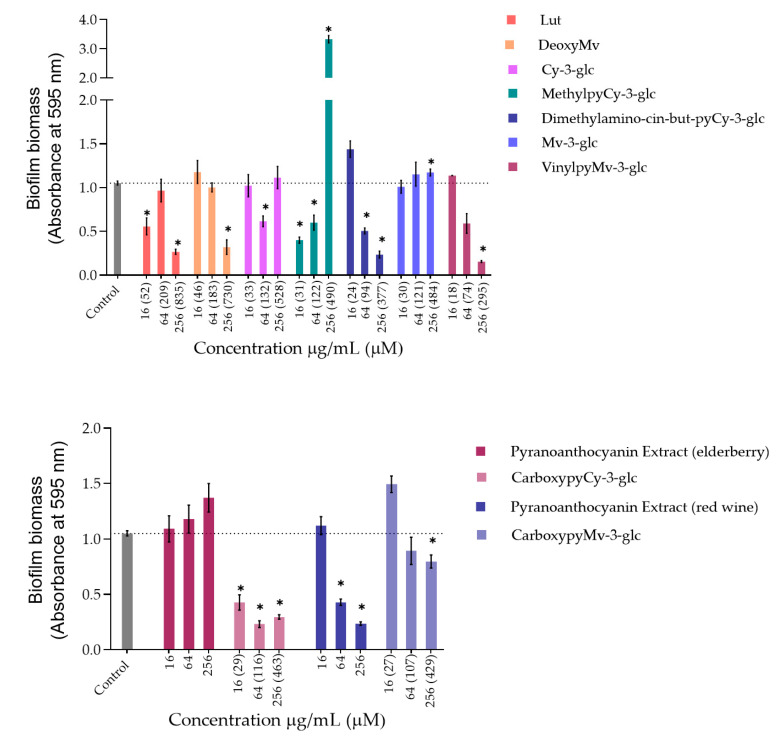
Biomass quantification of biofilms of *S. aureus* ATCC 29213 formed in the absence (control) and in the presence of different concentrations of extracts and compounds: 256 µg/mL, 64 µg/mL and 16 µg/mL. Statistically significant differences between biofilms formed in presence of extract/compound and control biofilm (*p* < 0.05) are marked with an asterisk.

Bacteria tend to naturally form biofilms, in which bacterial aggregates live encased in a matrix that consists of extracellular polymeric substances (EPS) produced by the bacteria [31]. The biofilm alternative lifestyle presents a self-organization, gene expression and growth rate that is entirely distinct from the planktonic (free-living) state. One of the main concerns about bacterial biofilms is their increased resistance to antimicrobial agents and difficulty of eradication. *P. aeruginosa* and *S. aureus* are some of the primary pathogens responsible for biofilm formation in chronic wounds. So, despite the lack of antibacterial activity exhibited by compounds and extracts, their ability to inhibit the biofilm formation of *P. aeruginosa* ATCC 27853 and *S. aureus* ATCC 29213 was evaluated through the crystal violet assay, and the results obtained are shown in Figure 2 and Figure 3, respectively.

Regarding *P. aeruginosa* biofilm biomass quantification in the presence of the compounds/extracts, it was possible to observe that at both concentrations of 64 and 256 µg/mL, only CarboxypyCy-3-glc significantly affected biofilm formation by *P. aeruginosa*, while at 64 µg/mL more compounds hampered biofilm formation in a significant manner, including deoxyMv, cy-3-glc, dimethylamino-cin-but-pyCy-3-glc and carboxypyMv-3-glc, as well as elderberry and red wine extracts. In some cases, however, an increase in the biofilm biomass was observed.

Concerning *S. aureus* biofilm formation, treatment with red wine extract B significantly affected biofilm formation at both 256 and 64 µg/mL, whereas elderberry extract did not present inhibitory activity at any of the concentrations tested. As can be observed, for most of the other compounds that interfered with biofilm formation, a reduction in biofilm biomass was only observed at one of those two concentrations. In the case of pyrano-derivatives of Cy-3-glc, carboxypyCy-3-glc was particularly efficient, significantly hampering biofilm formation at all three tested concentrations, while MethylpyCy-3-glc, despite clearly hampering biofilm formation at 16 and 64 µg/mL, led to the formation of a precipitate when tested at the highest concentration of 256 µg/mL that could not be removed during the biofilm staining protocol, resulting in a marked absorbance increase at 595 nm.

Furthermore, considering that CarboxypyCy-3-glc is the main component of elderberry extract and CarboxypyMv-3-glc is the major component of red wine extract, and confronting the results obtained for each extract with their main compound in both in *P. aeruginosa* and *S. aureus*, it was possible to draw some conclusions. In the case of CarboxypyCy-3-glc and elderberry extract, the isolated compound exhibited a much higher anti-biofilm activity. In fact, apart from the biomass reduction observed in *P. aeruginosa* when tested at a concentration of 64 µg/mL, elderberry extract did not show evidence of any inhibitory effects. This is possibly caused by antagonistic interactions between the different components of the extract, so that even with CarboxypyCy-3-glc being the major component of the extract (68% of the pyranoanthocyanins content) (Figure S1), its concentration is not enough to hamper biofilm formation and ends up being masked by the other elements: 12% are lipids (palmitic, oleic and stearic acids), 4% are formed by proteins and around 3% correspond to simple sugars (Table S1), which reinforces the importance of studying the compounds in their isolated form [32]. Comparing CarboxypyCy-3-glc with its anthocyanin analogue, Cy-3-glc, the extra pyran ring and carboxylic group appears to be a structural feature beneficial to its anti-biofilm activity. On the other hand, the inhibitory effect of red wine extract was more pronounced than its main component tested in an isolated form, CarboxypyMv-3-glc (considering the total pyranoanthocyanin content was only 30% CarboxypyMv-3-glc), which could indicate the occurrence of additive or synergistic interactions between different constituents: 10% proteins, 5% lipids (palmitic, oleic and stearic acids) and around 2% simple sugars (Table S1), contributing to the extract’s having the best performance (Figure S1). It is also worth mentioning that, besides the reduced phenolic content, the huge amount of polymeric phenolic structures likely present in this extract may account for the observed inhibitory effect on biofilm formation. After analysis of proanthocyanin content, no monomeric, dimeric or trimeric structures were identified, although anthocyanin derived polymeric structures may be present (not detected in any of the chromatographic or colorimetric assays). Besides, myricetin-*O*-(*O*-galloyl)arabinoside, myricetin-3-*O*-arabinoside, quercetin 3-methoxyhexoside and quercetin 3-*O*-glucuronide were tentatively identified by mass spectrometry and account for the flavonol content (Table S1), so they may have contributed to the observed effect too.

Most of these molecules impaired biofilm formation only at one or two of the concentrations tested; higher concentration did not mirror a higher inhibition; thus, it can be hypothesized that some of these agents impair biofilm formation at optimum concentrations, not in a dose-response manner [33]. Further, these results reinforce that the mechanism by which these compounds affect biofilm formation is not dependent on their ability to inhibit the bacterial growth but might be related with some interference with the quorum sensing system. In fact, the antibiofilm activities of numerous plant polyphenols, including flavonoids, phenolic acids and tannins, have been reported in previous studies (mostly conducted in *S. aureus*, *E. coli* and *P. aeruginosa*), where biofilm suppression was attributed to the capacity of these compounds to interfere with different bacterial regulatory mechanisms, including quorum sensing [34]. Therefore, it can be of interest to explore these compounds in a future work for their ability to interfere with the quorum sensing of *P. aeruginosa* as well as of *S. aureus*.

Considering that the vast majority of microbial and chronic infections are reported to be related to biofilm formation [35], these results provide a good indicator of the suitability of these compounds for managing biofilm-related diseases and their potential topical application could be an efficient way to hinder biofilm formation and the further development and persistence of infection.

### 3.2. Solar Protection Factor (SPF)

The use of sunscreens is one of the most widely used and efficient ways to protect the skin against UV-induced damage, by limiting the amount of radiation reaching the epidermal and dermal layers, either by reflecting and scattering (physical filters) or absorbing ultraviolet radiation (chemical filters). The presence of aromatic rings in their flavonoid core structure confers anthocyanins appreciable UV absorptive capacities, so it is no surprise that the application of these pigments has been reframed and proposed as natural UV-filters for photoprotective formulations [36,37].

As an initial screening test, each compound was prepared at a concentration of 0.2 mg/mL in ethanol and its corresponding SPF was determined according to the Mansur equation (Table 3). Overall, the obtained in vitro SPF values of anthocyanins and their structural derivatives support their UV filter activity and further application as additives in UV-protective formulations. Except for vinylpyranomalvidin-3-glucoside-catechin, which exhibited the lowest SPF (8.35), the general values ranged between approximately 14 to 30, with Lut exhibiting the highest SPF (29.82). Although not very marked differences were observed between the compounds, those belonging to the cyanidin group showed slightly higher SPFs compared to their structural analogues of the malvidin group, except for the methylpyranoanthocyanins which had similar values.

Oxybenzone, a commercially used chemical UV filter in numerous sunscreen formulations, exhibited a clearly superior UV-absorption capacity, exhibiting SPF values of 41.01 and 69.03, when tested at 0.1 and 0.2 mg/mL, respectively. Nevertheless, when the ethanolic solutions of anthocyanins and derivatives were supplemented with 0.1 mg/mL of oxybenzone, the estimated SPFs were close to that of oxybenzone alone (at 0.2 mg/mL). Overall, the SPF values obtained from the mixture between both agents (pigment + oxybenzone) suggest the occurrence of a cumulative effect. In the cases of MethylpyCy-3-glc and CarboxypyMv-3-glc, however, the resulting SPFs might be indicative of a potentiation effect arising from their combination with the commercial UV filter.

Oxybenzone, along with other conventionally used chemical UV filters such as octinoxate, continues to be allowed in the market (both in the US and EU), but the use of these ingredients remains a controversial topic of discussion. There seems to be no conclusive evidence about the prejudicial effects of oxybenzone to human health, although some reports have raised some concerns about their possible endocrine disruptor effects and photoallergic properties, along with the negative environmental impact regarding marine ecosystems [38,39]. Even though they might not completely replace the traditionally used physical and chemical UV filters, the use of these pigments could be an interesting option for enriching the formulations and allowing for a reduction of the required amounts of conventional filters to obtain a certain level of photoprotection. Additionally, possible synergism between these compounds and other filters could be an alternative strategy for reducing the formation of photodegradation byproducts and the possible phototoxic and photoallergic reactions caused by those reactive intermediates, as well as for preserving the SPF after UV irradiation [40]. This photostability effect has been described in previous studies [41]. To validate these promising yet preliminary results, incorporation of these ingredients in sunscreen formulations is required to understand if and to what extent they can increase SPF values and explore different combinations that could possibly provide synergistic photoprotective effects.

### 3.3. Cytotoxicity

**Figure 4:**
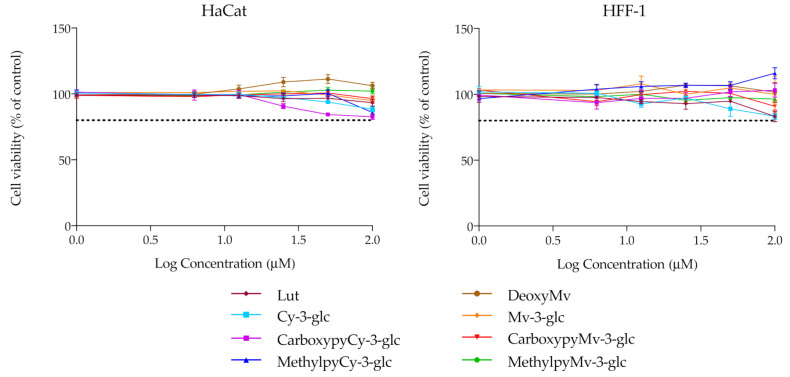
Cell viability of HaCat and HFF-1 cell lines in the presence of increasing concentrations of the compounds, evaluated by MTT assay. Dotted lines represent 80% viability.

For the remaining analysis, the group of compounds under study was narrowed, focusing merely on the native anthocyanins and their corresponding deoxyanthocyanins and pyranoanthocyanins. Before performing any other cellular experiments, their possible cytotoxic effects towards human epidermal keratinocytes and dermal fibroblasts were evaluated by MTT assay. Over a period of 48 h of incubation, none of the compounds had significant effects on the cellular viability of either of the two cell lines, up to 100 μM (Figure 4). Prior results with HaCat cells carried out with different anthocyanins and extracts have already demonstrated that Cy3glc has no significant cytotoxicity towards this cell line at concentrations below 100 μM [42]. In this study, pyrano derivatives and deoxyanthocyanins were also screened to identify potential cytotoxicity effects, but similarly to their anthocyanin counterparts, they appear to be safe and suitable for potential application in topical formulations.

### 3.4. Antioxidant Affect

**Figure 5:**
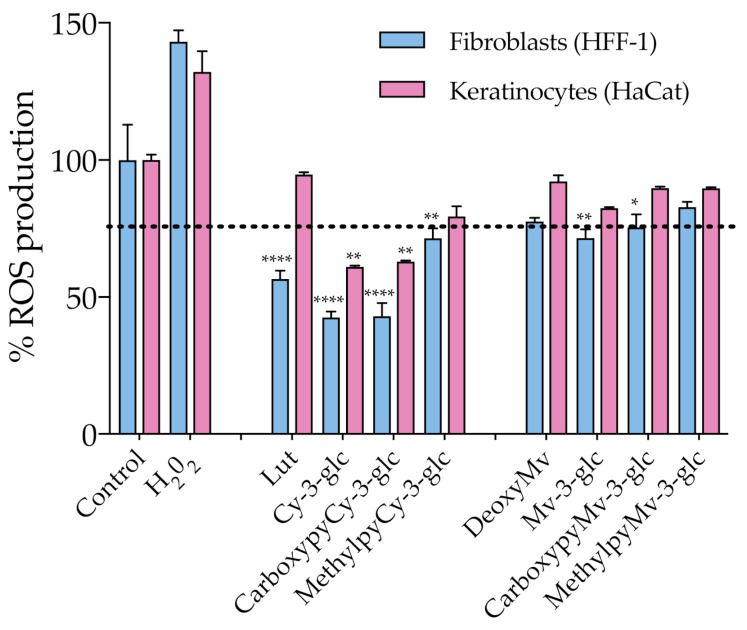
Effect of anthocyanins and related structures on ROS production in HaCat and HFF-1 cell lines, 24 h after incubation with 50 µM of each compound. Hydrogen peroxide (50 µM) was used as a positive control. Statistically significant differences between ROS production in the presence of the different compounds and control group are marked with asterisks: *p* < 0.05 (*), *p* < 0.005 (**) and *p* < 0.0001 (****). Dotted lines represent 80% ROS production.

As discussed earlier, oxidative stress induced by ROS overproduction is acknowledged as one of the most determinant events involved in the age-related decline of skin’s structural integrity and function. The use of antioxidants in skin care is one of the possible strategies to efficiently address and attenuate this process and consequent induced cellular and molecular events. With this in mind, ROS production within HaCat and HFF-1 cells was evaluated in the presence of the compounds at a fixed concentration of 50 μM, ensuring the absence of cytotoxicity (Figure 5). Results showed that Cy-3-glc related structures exhibited a more prominent effect on the reduction of the level of ROS, Cy-3-glc and CarboxypyCy-3-glc in particular, 24 h after incubation when compared to the control.

Apart from that, the antioxidant effect appears to be more pronounced in dermal fibroblasts than in epidermal keratinocytes. In an in vivo context, the proper function of the skin relies on the synergistic interactions established between both cell types, but the primary skin changes occur in the dermal section and aged fibroblasts are the main propagators, by paracrine mechanisms, of epidermal aging [43]. In fact, the oxidative stress environment, arising from both extrinsic and intrinsic sources, contributes to fibroblast collapse, which in turn amplifies the production of ROS, resulting in the simultaneous increased production of ECM degrading enzymes, reduced collagen synthesis and the elevation of multiple pro-inflammatory cytokines, creating an inflammatory microenvironment (commonly referred to as inflammaging) that has a major contribution to skin damage [6]. Therefore, even when considering that it is important to reinforce the antioxidant defense at the epidermal level (which is particularly susceptible to the damaging effects of acute UVR exposure), fibroblasts should be considered as one of the primary targets of antioxidant treatment. Topical application of antioxidants has the advantage of providing them directly to the specific site where their activity is required, supplementing the antioxidant system of the skin and reinforcing protection against oxidative stress. In the case of anthocyanins, besides their recognized ROS-scavenging capacity, several studies have reported their enhancing activity of endogenous antioxidant enzymes, including superoxide dismutase (SOD), catalase (CAT) and glutathione peroxidase (GPx), by stimulating their gene expression, which might have contributed to the observed antioxidant effect [44,45]. Considering the previously discussed UV-filter capacity of these compounds, a formulation containing them would benefit from a dual function of photoprotection. Even though anthocyanins appear to be appropriate ingredients for this type of application, undesirable color changes might occur under pH variations. On the other hand, pyranoanthocyanins exhibit a greater capacity to preserve their color intensity. In that regard, CarboxypyCy-3-glc, which exhibited similar antioxidant effects to Cy-3-glc, might provide an interesting option with regards to ensuring the long-term storage of a cosmetic formulation, given its enhanced structural stability compared to its anthocyanin counterpart.

### 3.5. Inhibition of Skin Aging Related Enzymes Activity

Hyaluronidase, collagenase and elastase constitute the three main enzymes responsible for the regulation of the structural integrity of the skin layers, being responsible for the degradation of hyaluronic acid, collagen, and elastin, respectively. Their exacerbated activity in the skin aging process and considerable potentiation by UVR exposure contribute to the progressive deterioration of the dermal connective tissue. For that reason, the activity of each of these skin degrading enzymes was monitored in the presence of the different compounds under study and compared to the activity of a control without the compound to determine whether they could reduce their activity. It should be mentioned that these enzymes display some structural differences from their corresponding human analogues. However, they are commonly used as model enzymes in studies, given their commercial availability in great amounts, providing a valuable tool for identifying potential inhibitors, particularly when screening a large number of compounds. The obtained results are summarized in Table 4.

In the case of elastase, the great majority of the tested compounds exhibited a negligible inhibitory effect, with the exception of Lut and MethylpyCy-3-glc, which presented a moderate rate of inhibition at 50 μM (27.1% and 23.7%, respectively). With respect to collagenase inhibition, both carboxypyranoanthocyanins stood out amongst the remaining compounds, resulting in an inhibition rate of 40%, which is significantly different behavior from that observed in the case of their methylpyrano counterparts, which revealed minor effects on the activity of the enzyme. Concerning hyaluronidase, apart from MethylpyMv-3-glc, all the compounds tested were able to interfere with the activity of the enzyme, exhibiting moderate inhibition rates ranging from 20 to 40%. Lut, CarboxypyCy-3-glc and Mv-3-glc exhibited higher inhibitory effects, reaching approximately 40%. When tested at higher concentrations (up to 200 μM), the inhibition rate increased to 64.5% in the case of Lut, 81.7% for CarboxypyCy-3-glc and 67.4% in the presence of Mv-3-glc (Figure S2) evidencing a dose dependent enzymatic inhibition.

Although the compounds were mostly tested at a fixed concentration of 50 μM in order to understand their relative inhibition potency within the specific set of conditions defined in this study, higher concentrations could and should be explored in the conception of topical formulations. The apparent absence of cytotoxicity issues at higher concentrations, evidenced by the above-mentioned cell viability results, supports the safety of these compounds as natural anti-aging components. It is also important to point out that in an in vivo context, topically administered compounds must cross the stratum corneum and the epidermal layer in order to reach the deeper skin layers, so higher doses should be considered in order to achieve the desired biological effects.

### 3.6. Molecular Docking Study with Hyaluronidase

Considering the overall better capacity of the compounds to modulate the activity of hyaluronidase, their interaction in the active site of the enzyme was also assessed by molecular docking. To validate the docking protocol, the X-ray tetrasaccharide was re-docked into the active site of the hyaluronidase, exhibiting a binding energy of −3.55 kcal/mol (Figure S3). Both X-ray and docking poses of the tetrasaccharide are very similar (root-mean-score deviation (RMSd) of 0.60 Å), and the main interactions with the enzyme were maintained. The tetrasaccharide established hydrophilic interactions with the Tyr55, Asp111, Glu113, Tyr184, Tyr227, Gln271, Ser303 and Ser304 residues as well as hydrophobic contacts with the Ile53 and Trp301.

Hyaluronidase catalyzes an acid/base reaction mechanism, in which the Glu113 acts as a proton donor and the acetamido group of hyaluronic acid acts as a nucleophilic base [25]. The relevance and the identification of the Glu113 as a catalytic residue is in line with studies of mutagenesis in the human sperm protein, hyaluronidase PH-20. Its substitution by a glutamine in the PH-20 protein resulted in a protein without activity, indicating that it is essential for the enzymatic reaction [46]. In addition, the aromatic triad (Tyr184, Tyr227 and Trp301) and the Asp111 and Gln271 residues were pointed out as relevant to keep the substrate in the appropriate position for catalysis (see Figure S4) [25]. Given that the alignment of the bee venom and human hyaluronidase sequences proved the conservation of the active site residues, it is possible to infer that they have similar catalytic mechanisms [25].

According to the literature, the hyaluronidase inhibition mechanism by phenolic compounds is competitive [26,47]. Therefore, the polyphenolic compounds used in the in vitro enzymatic inhibition assay were docked into the active site of the enzyme. Table S2 displays their binding energies (ΔG_binding_) and the nearest ligand group to the catalytic Glu113. Overall, compounds showed a good affinity for the enzyme (ΔG_binding_ between −4.17 and −8.06 kcal/mol), with their binding beng more favorable than the tetrasaccharide. These affinity values are similar to those previously suggested for several phenolic compounds (including Que-3-glc) from *Ravenala madagascariensis* (ΔG_binding_ between −4.02 and −7.12 kcal/mol) [26]. However, the order of affinity predicted by molecular docking is not in agreement with the order obtained in experimental kinetic assays, where it was observed that Que exhibited the highest percentage of inhibition among the tested compounds. Previous studies [26] also reported similar differences for related compounds targeting the hyaluronidase. Epicatechin, for example, showed a greater inhibition capacity than rutin (34.4% and 23.7%, respectively), whereas the results obtained from the molecular docking suggested the opposite (ΔG_binding_ for epicatechin and rutin were determined to be −4.85 and −7.12 kcal/mol, respectively). This fact might be due to the significant differences in molecular sizes, as larger compounds may establish more interactions with the enzyme, favoring their binding energies. However, other properties (e.g., charge polarization) may influence their inhibitory abilities. In this way, the modes of interaction of the various anthocyanins, as well as the main intermolecular interactions, namely with the catalytic Glu113, were analyzed, in order to understand the inhibitory activities obtained from the in vitro enzymatic experiments.

**Figure 6:**
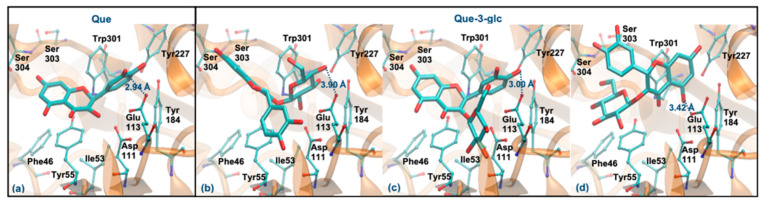
Representation of the structure of hyaluronidase complexed with Que (**a**) and Que-3-glc (interaction by glucose—(**b**), interaction by ring B—(**c**), interaction by rings A and C—(**d**)), showing the interacting residues of the active site. The enzyme is depicted in cartoon and colored in orange, the ligands are represented with sticks and colored by atom type, whilst the interacting residues are depicted in ball-and-sticks and colored by atom type. Hydrogen atoms are not represented to simplify the visualization.

As mentioned, Que and Que-3-glc are considered good hyaluronidase inhibitors [26]. However, their docking binding affinities are slightly lower than those obtained for the other compounds (which can be due to the error range expected for this method) [48]. Figure 6 displays the best docking poses for the two molecules. Que has a ΔG_binding_ of −5.94 kcal/mol, interacting via its ring B (Figure 6a), in agreement with previous studies that predicted a ΔG_binding_ of −5.15 kcal/mol and a similar interaction mode [49]. Like the tetrasaccharide, Que interacts with Glu113 at a distance of less than 3 Å. In addition, it establishes hydrogen interactions with Asp111, Tyr184, Ser304 and Asp305 residues and interacts by stacking π-π with the Tyr55 and Trp301 residues. Regarding the Que-3-glc, it can interact with hyaluronidase by different moieties (glucose, ring B or rings A–C). Although binding through the glucose slightly favors the affinity, in this situation the ligand is further away from Glu113 and there is a loss of the π-π stacking with Tyr55 (Figure 6b). The interaction through ring B is very similar to the Que (Figure 6c). The interaction mode by rings A–C is in agreement with those obtained in the literature [26] (Figure 6d). The proximity to the catalytic Glu113 is largely favored when the interaction occurs via the B ring. Based on this, the binding of the Que-3-glc should be similar to the respective aglycone (please see Figure 6c).

**Figure 7:**
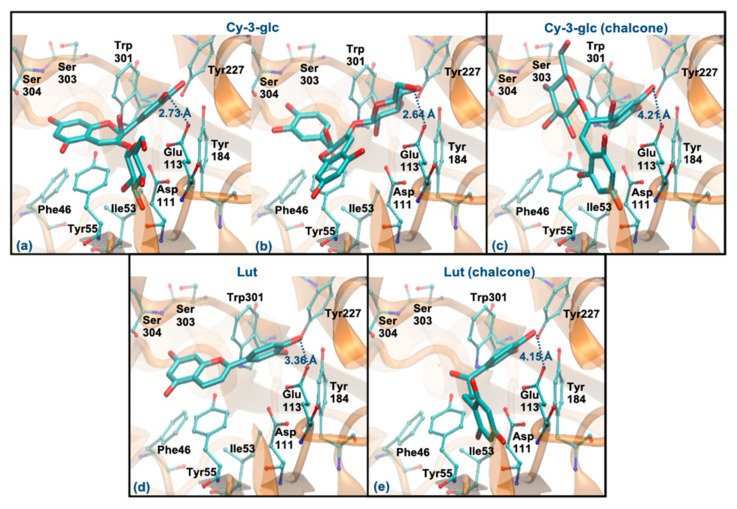
Representation of the structure of hyaluronidase complexed with Cy-3-glc—pseudobase carbinol (interaction by ring B—(**a**), interaction by glucose—(**b**)) and chalcone (interaction by ring B—(**c**))—and Lut–pseudobase carbinol (interaction by ring B—(**d**)) and chalcone (interaction by ring B—(**e**))—showing the interacting residues of the active site. The enzyme is depicted in cartoon and colored in orange, the ligands are represented with sticks and colored by atom type, whilst the interacting residues are depicted in ball-and-sticks and colored by atom type. Hydrogen atoms are not represented to simplify the visualization.

Cy-3-glc (pseudobase carbinol) and Lut (pseudobase carbinol) have the greatest affinities for the enzyme when interacting through the ring B. In fact, the interaction mode of ring B and the main intermolecular interactions of Cy-3-glc are very similar to those of Que (Figure 7a). Curiously, when it interacts through the glucose, the distance to Glu113 and the main interactions are quite similar (Figure 7b). In the form of chalcone, Cy-3-glc has a lower affinity for the enzyme. This structure interacts through ring B, but it is much further away from Glu113 (distance > 4 Å) and loses its π-π stacking with the Tyr55 (Figure 7c). Lut also interacts through the ring B, in a very similar way to Que. However, it slightly deviates from Glu113, and reduces the interaction with Asp111 due to the absence of any substituent on C3 (hydroxyl group or glucose unit) (Figure 7d). The chalcone form of Lut binds much further away from Glu113 (distance > 4 Å) and Tyr55 and does not interact with the Ser303, Ser304 and Asp305 residues (Figure 7e). The breaking of the C ring on the chalcone forms could be responsible for their weaker binding because it allows higher freedom of movement, which may reduce interaction with the aromatic residues of the enzyme. These results agree with those obtained experimentally, assuming that the majority forms in solution are those of the pseudobase carbinol, since a high percentage of inhibition was observed for these compounds.

**Figure 8:**
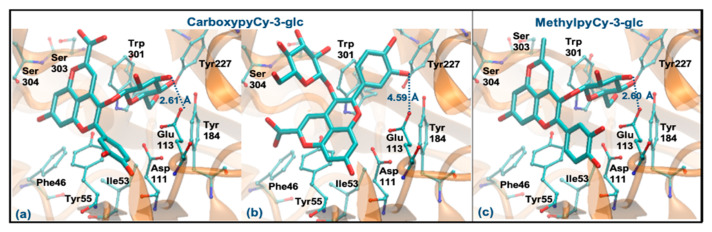
Representation of the structure of hyaluronidase complexed with CarboxypyCy-3-glc (interaction by glucose—(**a**), interaction by ring B—(**b**)) and MethylpyCy-3-glc ((interaction by glucose—(**c**)), showing the interacting residues of the active site. The enzyme is depicted in cartoon and colored in orange, the ligands are represented with sticks and colored by atom type, whilst the interacting residues are depicted in ball-and-sticks and colored by atom type. Hydrogen atoms are not represented to simplify the visualization.

Regarding CarboxypyCy-3-glc, it can interact through the glucose or via its ring B. However, the former binding mode is plainly favored (Δ_Gbinding_ of −8.06 kcal/mol vs. −6.06 kcal/mol and distance to Glu113 of 2.61 Å vs. 4.59 Å). Intermolecular interactions with the key active site residues are maintained. Despite the ligand being slightly further away from Tyr55, it made a perpendicular stacking with the pyran group (Figure 8a). Oppositely, when it interacted through the ring B, the parallel stacking with Tyr55 was strongly favored, which can support a competition between the two different interaction modes (see Figure 8b). MethylpyCy-3-glc interacts by its glucose unit (ΔG_binding_ of −7.15 kcal/mol) and despite its proximity to Glu113 (distance < 3 Å), the remaining groups of the compound bind in a shallow mode, reducing the interactions with the enzyme (Figure 8c).

**Figure 9:**
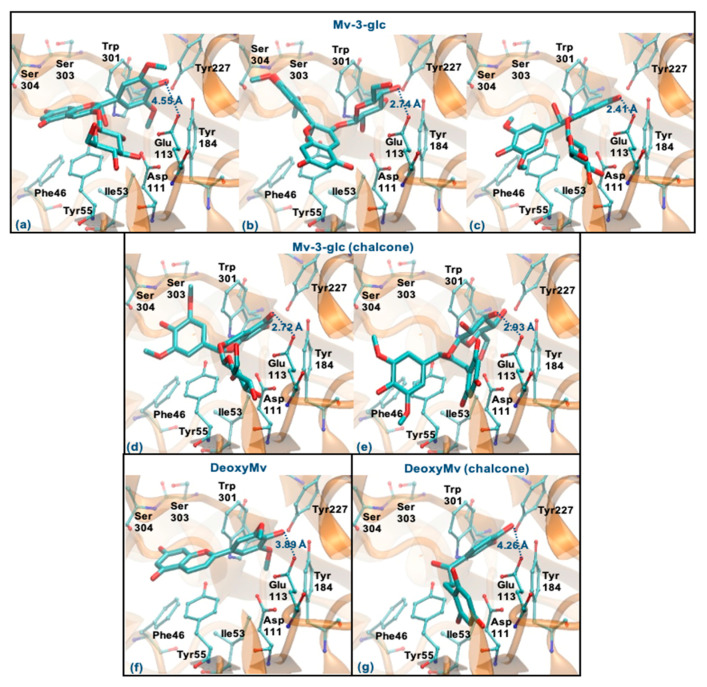
Representation of the structure of hyaluronidase complexed with Mv-3-glc—pseudobase carbinol (interaction by ring B—(**a**), interaction by glucose—(**b**), interaction by rings A and C—(**c**)) and chalcone (interaction by rings A and C—(**d**), interaction by glucose—(**e**))—and DeoxyMv–pseudobase carbinol (interaction by ring B—(**f**)) and chalcone (interaction by ring B—(**g**))—showing the interacting residues of the active site. The enzyme is depicted in cartoon and colored in orange, the ligands are represented with sticks and colored by atom type, whilst the interacting residues are depicted in ball-and-sticks and colored by atom type. Hydrogen atoms are not represented to simplify the visualization.

Mv-3-glc (pseudobase carbinol) can interact with the enzyme by the ring B, glucose or rings A-C. The former binding mode is similar to Que and Cy-3-glc, however, the presence of methoxy groups in ring B hinders the proximity to Glu113 (staying at a distance of 4.55 Å) (Figure 9a). The two latter binding modes clearly favor proximity to Glu113 (distance < 3 Å), the parallel stacking with Tyr55 and/or the perpendicular π-π stacking with Trp301 and Tyr227 as well as dispersive/hydrophobic contacts favored by the presence of methoxy groups (Figure 9b,c). In the form of chalcone, it can interact by the rings A and C or by glucose (in a similar way to those described for Mv-3-glc). These binding modes may be favored, which justify the good inhibitory capacity of this compound (Figure 9d,e). Analyzing the results obtained for DeoxyMv, the mode of interaction by ring B is very similar to that described for Mv-3-glc and for Lut. However, the presence of methoxy groups in ring B hinders proximity to Glu113 (distance of 3.89 Å) (Figure 9f). The same is observed for chalcone (distance of 4.26 Å) (Figure 9g). This higher distance to Glu113 (+0.5 Å) may be responsible for the lower inhibitory activity of this compound.

**Figure 10:**
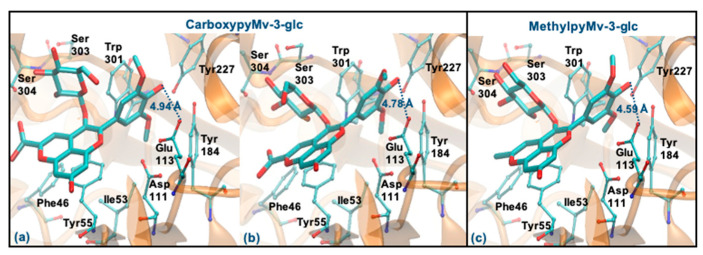
Representation of the structure of hyaluronidase complexed with CarboxypyMv-3-glc (interaction by ring B—(**a**,**b**)) and MethylpyMv-3-glc (interaction by ring B—(**c**)), showing the interacting residues of the active site. The enzyme is depicted in cartoon and colored in orange, the ligands are represented with sticks and colored by atom type, whilst the interacting residues are depicted in ball-and-sticks and colored by atom type. Hydrogen atoms are not represented to simplify the visualization.

Regarding the two pyrano derivatives of Mv-3-glc, CarboxypyMv-3-glc and MethylpyMv-3-glc, these compounds interact by ring B, with ΔG_binding_ values between −6.77 and −6.15 kcal/mol. These molecules are docked far away from Glu113 (distances > 4 Å), probably due to the presence of methoxy groups in the ring B. In addition, as the bulky pyrano group binds in a shallow mode, it is much further away from the Asp111, Tyr227, Tyr265 and Trp301 residues (Figure 10a–c).

Overall, the structural analysis of the docking data suggests that the interactions of anthocyanins with residues Tyr55, Glu113, Tyr184 and Trp301 are determinant of their inhibitory capacity. In addition, contacts with residues Asp111 and Tyr227 are important, but not essential (evidenced, for example, by the interaction mode of the potent inhibitor Lut). The binding of the compounds is plainly favored by a hydrophilic interaction with the catalytic Glu113 (that can occur via ring B, rings A and C or the glucose unit of Cy-3-glc derivatives), as well as by a narrow π-π stacking with Tyr55 and Trp301 residues.

### 3.7. Transport Efficiency

Despite the beneficial effects that the potential topical application of these compounds might offer, as suggested by the results presented so far, the efficiency of such treatment ultimately depends on the capacity of these bioactives to overcome the stratum corneum barrier for permeation across the skin. However, penetration and permeation studies about these types of compounds are still scarce.

**Figure 11:**
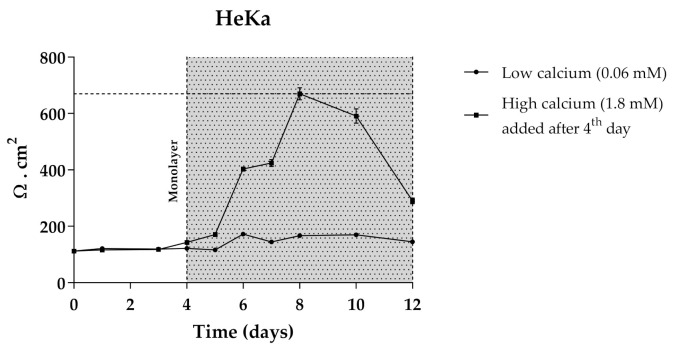
TEER measurements of HeKa cell monolayer during 12 days of culture. Cells were kept for 4 days in low calcium medium (0.06 mM) to allow monolayer formation. For differentiation, in the last 8 days medium was changed to high calcium (1.8 mM).

The use of 3D skin models, which faithfully mimic the in vivo epidermis and skin barrier function has emerged as a useful alternative to animal testing. Besides the complex experimental conditions, the large quantities of primary keratinocytes required represent limitations for their application as screening models. The immortalized keratinocyte cell line HaCat could be a solution for overcoming these issues. The HaCaT cell line is widely used in keratinocyte monolayer culture models [50] and responds to differentiation-promoting stimuli, such as contact inhibition and high calcium concentrations in the culture medium [51], but the transcriptional expression pattern of cornified envelope-associated proteins, such as filaggrin, loricrin and involucrin is abnormal compared to normal human primary keratinocytes [52]. Considering this, primary normal human epidermal keratinocytes (Heka) were chosen to develop the optimal conditions of cellular differentiation and create a proper skin barrier model. Given the overall good performance of CarboxyCy-3-glc, it was selected for the transport experiments as a representative compound of the anthocyanin derivatives, along with its corresponding native anthocyanin, Cy-3-glc, in order to investigate how their structural differences might affect the transport profile. Cells were grown in low calcium medium (0.06 mM) until reaching confluence. At the 4th day of culture, medium was switched to high calcium (1.8 mM). Only Heka monolayers with an integrity equivalent to a transepithelial electrical resistance (TEER) higher than 450 Ω.cm^−2^ were used for the transport studies. These values were reached after a total of 8 days in culture (Figure 11).

**Figure 12:**
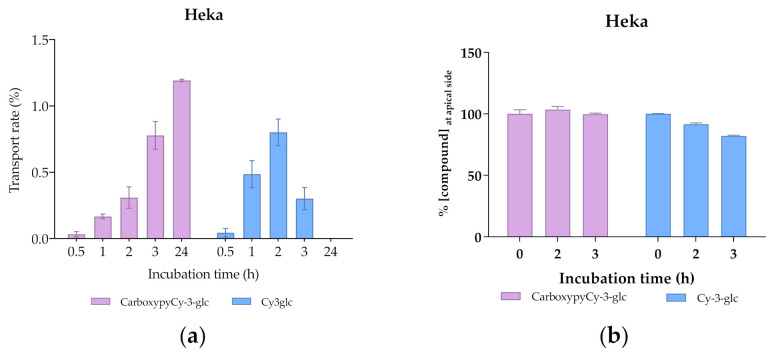
Transport efficiency of 200 µM of Cy3glc and CarboxypyCy-3-glc through HeKa cells (apical → basolateral) monitored for 24 h at the (**a**) basolateral and (**b**) apical side.

Comparing the transport profile of both compounds in the first 2 h of culture, transport appears to be slightly slower in the case of CarboxypyCy-3-glc, which could be due to its higher structural complexity compared to Cy-3-glc (Figure 12a). However, throughout the 24 h period, a continuous increase in the amount of the carboxypyrano derivative transported across the barrier could be detected, contrary to what was observed for Cy-3-glc, since the transport rate decreased after 2 h in culture. A reduction in the apical amount of Cy-3-glc available for uptake was observed but does not justify the reduction in the transport efficiency detected in the basolateral side after 3 h (Figure 12). In fact, this apparent reduction in the transport efficiency of Cy-3-glc can be explained by its lower stability in comparison with its corresponding carboxypyrano derivative in pH and temperature conditions (Figure 12b). In this system, the cell culture medium lacks the presence of fetal bovine serum, which eliminates the influence of the enzymatic machinery on the stability of the compounds. In fact, previous research on the stability of anthocyanins in cell culture medium showed that the concentration of Cy-3-glc decreased after 3 h of incubation, from 100 µM to 88.0 µM [53].

The observed uptake of both Cy-3-glc and CarboxypyCy-3-glc may result from the involvement of glucose transporters, namely GLUT1, 2, 3 and 5 expressed in keratinocytes [54], as previously described for other barrier models [55].

The greater molecular weight of CarboxypyCy-3-glc did not pose a constraint on its transport across the cellular barrier. Interestingly, a recent analysis concerning the release of anthocyanins from a lipophilic formulation and their skin penetration capacity through the stratum corneum, carried out in both porcine and human skin samples, revealed that anthocyanins from both elderberry (with molecular weights ranging from 449 to 581 Da) and red radish (933–1019 Da) successfully penetrated into the skin. Pigments were found to reach relevant depths to exert their biological activity, indicating that the higher molecular weight of red radish anthocyanins did not prevent their diffusion across the skin [56].

## 4. Conclusions

Overall, the results presented herein shed some light on some of the skin-health promoting effects of this group of pigments through distinct levels of action. Topical routes of administration of these compounds have been an increasingly explored topic, providing a targeted and apparently safe therapy. However, to ensure the effectiveness of their biological activities, different parts of the equation must be taken into consideration when conceiving a topical formulation, including the properties of the compounds and of the formulation in which they are incorporated, which will determine their capacity to overcome the stratum corneum. Further tests focused on the skin penetration behavior of these compounds and their quantification within the skin layers are crucial to determine the required doses to achieve the expected benefits in vivo and guide the optimization process of concentrations and compounds ratios.

Furthermore, the use of natural extracts rather than isolated compounds is commonly claimed to be more effective due to the potential beneficial synergistic interactions between their different constituents. However, the opposite effect, antagonism, should also be considered, as the effects of active agents might be masked by other compounds present in the same mixture, as demonstrated in the results regarding the inhibition of biofilm formation. Therefore, the study of highly purified compounds, although representing a time-consuming process, is an important tool for evaluating and comprehending the full potential of each molecule in terms of its biological effects.

Although anthocyanins and anthocyanin-rich sources have long provided solid evidence of their skin-protective properties, their applicability is constantly haunted by stability issues. The inclusion of anthocyanin structural derivatives represents an appealing alternative to overcome this issue. Among all anthocyanin-related structures tested, Carboxypyranocyanidin-3-*O*-glucoside in particular appeared to be a promising candidate given its overall good performance in the different staged experiments and its higher structural stability.

## 5. Patents

Provisional patent application N. 117058: Process for extraction and hemi-synthesis of pyranoanthocyanins and skincare cosmetic formulations containing them.

## Figures and Tables

**Table 1:** Normalized product function used for the calculation of SPF.

λ (nm)	EE × I (Normalized)
290	0.0150
295	0.0817
300	0.2874
305	0.3278
310	0.1864
315	0.0839
320	0.0180
Total	1

EE—erythemal effect spectrum; I—solar intensity spectrum.

**Table 2:** Minimum inhibitory concentration (MIC) and minimum bactericidal concentration (MBC) values, in µg/mL and in mM, of compounds and extracts against *P. aeruginosa* ATCC 27853, *S. aureus* ATCC 29213, *M. luteus* ATCC 4698, *S. epidermidis* ATCC 14990 and *S. pyogenes* ATCC 19615.

Extract/Compound	*P. aeruginosa* ATCC 27853	*S. aureus* ATCC 29213	*M. luteus* ATCC 4698	*S. epidermidis* ATCC 14990	*S. pyogenes* ATCC 19615
	MIC—µg/mL (mM)				
Lut	>512 (>1.67)	>512 (>1.67)	128 (0.42) *	128 (0.42) *	>512 (>1.67)
DeoxyMv	>512 (>1.46)	>512 (>1.46)	>512 (>1.46)	>512 (>1.46)	>512 (>1.46)
Cy-3-glc	>512 (>1.06)	>512 (>1.06)	>512 (>1.06)	512 (1.06) *	>512 (>1.06)
CarboxypyCy-3-glc	>512 (>0.93)	>512 (>0.93)	>512 (>0.93)	>512 (>0.93)	>512 (>0.93)
Elderberry carboxypyranoanthocyanin extract (Elderberry extract)	>512	>512	512 *	>512	>512
MethylpyCy-3-glc	>512 (>0.98)	>512 (>0.98)	>512 (>0.98)	>512 (>0.98)	>512 (>0.98)
Dimethylamino-cin-but-pyCy-3-glc	>512 (>0.75)	>512 (>0.75)	64 (0.09) *	>512 (>0.75)	512 (0.75) *
Mv-3-glc	>512 (>0.97)	>512 (>0.97)	>512 (>0.97)	>512 (>0.97)	>512 (>0.97)
CarboxypyMv-3-glc	>512 (>0.86)	>512 (>0.86)	>512 (>0.86)	>512 (>0.86)	>512 (>0.86)
Red wine carboxypyranoanthocyanin extract (red wine extract)	>512	>512	512 *	>512	>512
VinylpyMv-3-glc-catechin	>512 (>0.59)	>512 (>0.59)	>512 (>0.59)	>512 (>0.59)	>512 (>0.59)

* Only in these cases, the MBC assay could be performed, yet, MBC was >512 µg/mL.

**Table 3:** In vitro solar protection factors (SPF) of anthocyanins and derivatives estimated in the absence and presence of oxybenzone.

Compound (0.2 mg/mL)	SPF	
	-	+ 0.1 mg Oxybenzone/mL
Lut	29.82 ± 0.02	71.89 ± 0.040
DeoxyMv	17.28 ± 0.03	55.25 ± 0.03
Cy-3-glc	22.38 ± 0.01	63.01 ± 0.02
Blackberry anthocyanin extract	20.10 ± 0.06	66.98 ± 0.03
CarboxypyCy-3-glc	20.71 ± 0.18	n.d.
Elderberry carboxypyranoanthocyanin extract	16.03 ± 0.01	64.36 ± 0.03
MethylpyCy-3-glc	18.53 ± 0.15	68.21 ± 0.08
Dimethylamino-cin-but-pyCy-3-glc	14.78 ± 0.02	59.69 ± 0.01
Mv-3-glc	20.96 ± 0.01	n.d.
CarboxypyMv-3-glc	13.92 ± 0.04	67.85 ± 0.04
Red wine carboxypyranoanthocyanin extract	21.60 ± 0.02	67.10 ± 0.02
MethylpyMv-3-glc	19.24 ± 0.01	56.85 ± 0.06
VinylpyMv-3-glc-catechin	8.35 ± 0.06	n.d.
-		41.01 ± 0.38
Oxybenzone	69.03 ± 0.17	

n.d.—not determined.

**Table 4:** Inhibitory activities of anthocyanins and related structures against hyaluronidase, collagenase and elastase. Quercetin at 50 μM, epigallocatechin gallate (EGCG) at 50 μM and N-(Methoxysuccinyl)-Ala-Ala-Pro-Val-chloromethyl ketone (MAAPVCK) at 10 μM were used as positive controls for hyaluronidase, collagenase and elastase, respectively. Results are presented as the mean ± standard error deviation (SEM) of at least 3 independent experiments.

	Hyaluronidase	Collagenase	Elastase
Compound	% Inhibition (50 μM)		
Lut	40.1 ± 2.91	24.2 ± 2.95	27.1 ± 2.71
Cy-3-glc	31.7 ± 4.18	28.5 ± 2.69	13.4 ± 2.57
CarboxypyCy-3-glc	38.1 ± 3.56	40.4 ± 2.59	5.45 ± 1.81
MethylpyCy-3-glc	17.6 ± 1.67	7.33 ± 2.47	23.7 ± 1.87
DeoxyMv	21.8 ± 2.59	n.a.	n.a.
Mv-3-glc	40.8 ± 1.33	n.a.	5.01 ± 3.16
CarboxypyMv-3-glc	28.1 ± 2.67	40.5 ± 4.31	3.54 ± 5.51
MethylpyMv-3-glc	1.92 ± 0.467	8.04 ± 4.48	5.65 ± 5.37
Quercetin	77.6 ± 2.28	n.d.	n.d.
EGCG	n.d.	86.7 ± 5.31	n.d.
MAAPVCK	n.d.	n.d.	94.6 ± 0.759

n.a.—not active; n.d.—not determined.

## Data Availability

Data is contained within the article and supplementary material.

## Supplementary Materials

The following are available online at https://www.mdpi.com/article/10.3390/antiox10071038/s1, Figure S1: Chromatographic profile of (a) blackberry anthocyanins, (b) elderberry and (c) red wine pyranoanthocyanins, at 520 nm. Figure S2: Hyaluronidase inhibition rates in the presence of increasing concentrations of Lut, CarboxypyCy-3-glc and Mv-3-glc. Figure S3: Superimposition of the docking and crystallographic geometries of the tetrasaccharide bound into the active site of the hyaluronidase. Enzyme is depicted in cartoon and colored in orange, while the tetrasaccharide is represented with sticks and colored in blue (docking pose) or orange (X-ray pose). Hydrogen atoms are not represented to simplify the visualization, Figure S4: Representation of the structure of hyaluronidase complexed with tetrasaccharide, showing the interacting residues of the active site. The arrow is pointing to the atom where the cleavage occurs. The enzyme is depicted in cartoon and colored in orange, the tetrasaccharide is represented with sticks and colored by atom type, whilst the interacting residues are depicted in ball-and-sticks and colored by atom type. Hydrogen atoms are not represented to simplify the visualization. Table S1: Chemical composition and antioxidant activity of the anthocyanin and pyranoanthocyanin extracts. Table S2: Values of ΔG_binding_ for the best molecular docking solutions obtained for each compound. The closest group to Glu113 was also identified as well as the respective distance between oxygen atoms. The interacting residues of the active site were identified.
