# Supplementary material for: Anthocyanin-Related Pigments: Natural Allies for Skin Health Maintenance and Protection
Source: Antioxidants (Basel). 2021 Jun 28;10(7):1038. doi: 10.3390/antiox10071038 (PMC8300807; doi:10.3390/antiox10071038)
Supplement: Supplementary file 1 [file antioxidants-10-01038-s001.zip › antioxidants-1282622-supplementary.pdf]

Supplementary Materials and Methods

- **Characterization of extracts**

High-performance liquid chromatography with diode-array detection (HPLC-DAD) analysis

Anthocyanin and pyranoanthocyanin extracts were analyzed by reverse phase liquid chromatography (Dionex Ultimate 3000, Thermo Scientific) on a C18 gel column (250 mm x 4.6 mm (i.d.); 5 µm, Thermo Scientific). Solvents used were (A) water/formic acid 9/1 (v/v) and (B) water/acetonitrile/formic acid 6/3/1 (v/v/v), with the following gradient: 80-15 % A and 20-85 % B over 70 minutes, 15-0 % A and 85-100 % B from 70 to 70.1 minutes, with a flow rate of 1.0 mL/minute. Column was washed with 100 % B for 10 minutes and then stabilized under the initial conditions for 10 minutes. Detection was carried out between the 200 and 800 nm, with a DAD detector. Pure cyanidin-3-*O*-glucoside and CarboxypyCy-3-glc were prepared in the range of 0.125-0.750 mg/mL (0.258-1.55 mM) and used as standards for the calibration curves.

To determine the content of low molecular weight polyphenols and flavonols, extracts were mixed with ethyl acetate and acetonitrile 2/2/1 (v/v/v) in microtubes, placed in a shaker for 10 seconds and centrifuged for 5 minutes at 8000 rpm. After centrifugation and phase separation, liquid-liquid extraction was repeated for the aqueous phase. Organic phases were combined and the organic solvent evaporated by speed vacuum. The obtained fraction was then re-suspended in water/methanol 1/1 (v/v).

Content of low molecular weight polyphenols in all extracts was determined by reverse phase liquid chromatography (Dionex Ultimate 3000, Thermo Scientific) on a C18 gel column (150 mm × 4.6 mm (i.d.); 5 µm, Thermo Scientific). Solvents used were (A) water/acetic acid 99/1 (v/v) and (B) water/acetonitrile/acetic acid 79/20/1 (v/v/v), with the following gradient: 80-20% A and 20-80% B for 55 minutes, 20-10% A and 80-90% B from 55 to 70 minutes, 10-0% A and 90-100% B from 70 at 90 minutes, at a flow rate of 0.3 mL/minute, as previously reported [1]. Column was washed with 100% B for 10 minutes and then stabilized under the initial conditions for 15 minutes. Detection was performed using a DAD detector and it was carried out between 200 and 800 nm. Calibration curve was obtained using gallic acid as standard, in a concentration range of 0.0001 to 0.1701 mg/mL (0.590-999 µM) (r^2 = 0.9999).

Content of proanthocyanidins in red wine carboxypyranoanthocyanin extract (red wine extract) was determined by HPLC-DAD analyses in a Jasco LC-4000 HPLC system equipped with an Agilent Poroshell 120, C18 reverse-phase column (250×4.6 mm i.d., 2.7 µm) thermostated at 25°C. The mobile phase was composed by solvent A, 0.1% (v/v) formic acid aqueous solution, and solvent B, 0.1% (v/v) formic acid in HPLC grade acetonitrile; linear gradient (0 min, 10% eluent B to 50 min, 28% eluent B); flow rate of 0.5 mL min^{-1}. Spectra were recorded from 220 to 600 nm, and detection was carried out at 280 nm as the preferred wavelength using a Photo diode array detector (MD-4010). The compounds were identified based on the mass spectra data, retention time of standards and/or comparison with the literature [2].

Concentration of flavonols in the extracts was determined according to the experimental conditions described in the literature [3], with slight modifications. Samples were analyzed by reverse phase liquid chromatography (Dionex Ultimate 3000, Thermo Scientific) on a C18 gel column (150 mm × 4.6 mm (i.d.); 5 µm, Thermo Scientific). Solvents used were (A) water/formic acid 99/1 (V/V) and (B) acetonitrile, with the following gradient: 90-50% A and 10-50% B over 50 minutes, 50-0 % A and 50-100% B from 50 to 70 minutes, 0% A and 100% B for 10 minutes and then stabilized under the initial conditions for 10 minutes, with a flow rate of 0.5 mL/minute. Detection was performed using a DAD detector and carried out between 200 and 800 nm. A calibration curve was obtained using quercetin-3-galactoside as standard in a concentration range of 0.050 to 0.200 mg/mL (0.11-0.43 mM) (r^2 = 0.9933).

High performance liquid chromatography with mass spectrometry (HPLC-MS) analysis

Anthocyanin and pyranoanthocyanin extracts were characterized according to previously described experimental conditions [1,3,4], with some adaptations. Finnigan Surveyor HPLC equipped with a C18 reverse phase column (150 mm × 4.6 mm (i.d.); 5 µm, Hypersil Gold, Thermo

Finnigan) thermostated at 25 °C. The same solvents were used, under the same conditions, as in the HPLC analysis described in section 2.3.1.) with the exception of the concentration of formic acid that was only 1% in solvents A and B. This equipment was coupled to a LCQ DECA XP MAX mass detector (Finnigan Corp.), equipped with an atmospheric pressure ionization source (API), using electrospray ionization (ESI) source. The vaporizer and capillary voltages were 5 kV and 4.0 V, respectively. The capillary temperature was 325 °C. Nitrogen was used as a coating gas (or nebulizer) and as an auxiliary gas with flows of 40 and 15 (arbitrary units), respectively. The detection was made by spectrophotometry with a photodiode and by MS. Spectra were obtained in positive and negative modes with the range of *m/z* between 100 and 2000. The mass spectrometer was programmed to make a series of scans: "full mass" (MS), a zoom scan of the most intense ion (MS^2) and a MS-MS analysis of the most intense ion using the relative collision energy of 45 (MS^3).

Determination of protein, sugar and lipid content

Protein content in three extracts was determined using the Bradford assay, as described in the literature [5], with some modifications. Standard BSA solutions were prepared in the range of 0.010 to 0.050 mg/mL, in triplicate, to obtain the calibration curve (r^2 = 0.9988).

In 96-well plates, 100 µL of each aqueous solution of (pyrano)anthocyanin extract or standard BSA solution were mixed with 200 µL of Bradford's reagent. Sample was replaced by distilled water for the blank preparation. Plate was left at room temperature for 10 minutes, protected from light Absorbance at 595 nm was then measured on a plate reader (Biotek Powerwave XS).

Sugar content in each extract (1 mg/mL in water) was determined by GC-MS procedure [6]. Derivatization with hexamethyldisilazane (HMDS) was applied for the trimethylslylation (TMS) of polar functional groups. The procedure was as follows: (a) in the first step, 600µL of HMDS + acetonitrile mixture (1:1 v/v) was added as a silylation agent to 20µL of aqueous sample for the derivatization of easily silylable functional groups (e.g., hydroxyl in glucose), 2µL of trifluoroacetic acid was added as a catalyst and the sample was heated to 50◦C for 30 min. The vial was left open during this process to ensure the escape of the ammonia gas produced in the reaction. Subsequently, (b) in the second step, 400µL of pure HMDS was added and the mixture was heated to 80◦C for 30 min in a closed vial. After cooling to laboratory temperature 1µL of the resulting solution was injected into GC–MS/MS system.

The GC–MS/MS analyses were carried out with a Trace 1300 gas chromatograph equipped with a split–splitless injector, a autosampler 1310 Thermo Scientific and a ISQ Single quadrupole MS (Thermo Fisher, Austin, TX, USA). A total of 1 µL of the sample was injected into the injector operating in splitless mode. The temperatures of the injector and the MS-transfer line were 250 ◦C and 300 ◦C, respectively. Compounds were separated on a 30 m × 0.25 mm (i.d.) × 0.25 µm DB-17 capillary column (Agilent Technologies, CA, USA) operating at constant helium flow of 1.5 mLmin−1. The column temperature was initially set to 110 ◦C, held for 5 min, which increased at a rate of 6 ◦Cmin−1 to 300 ◦C held for 5 min. Measurements were performed in SCAN mode with m/z range set to 40 −1100. The MS conditions were as follows: ion source temperature 280 ◦C and electron energy 70 eV. Selected ion monitoring (SIM) conditions were used for the glucose, selecting the m/z 204.

Lipid content in each extract (1 mg/mL in water) was determined by GC-MS procedure. Briefly the extracts were subjected to a liquid-liquid extraction with chloroform and evaporated to dryness. To the remaining solid 1 mL of 0.5M KOH solution in methanol was added and 2 mL of BF_3 solution in methanol. The mixture was transferred to a hydrolysis tube and the tube closed tightly. The tubes were heated in a water bath for 10 minutes (in the hood) and then cooled on ice. After addition of ethyl ether, the solution was subjected to a liquid-liquid extraction with water. The water content in the organic layer was removed by the addition of enough anhydrous sodium sulfate. Samples were analyzed by GC-MS according to the procedure above, with slightly modifications: ion source and MS-transfer line temperatures were 280 ◦C and 250 ◦C, respectively; palmitic acid was used as standard for the calibration curve. The column temperature

was initially set to 110 ◦C, held for 2 min, until 185 ◦C, which increased at a rate of 30 ◦C.min^{-1} (during 2 min) until 205 ◦C, held for 5 min, which increased at a rate of 30 ◦C.min^{-1} until 250 ◦C, held for 10 min, which increased at a rate of 30 ◦C.min^{-1} until 280 ◦C, held for 10 min.

Determination of antioxidant and antiradical activity

Ferric Reducing Antioxidant Power (FRAP) assay was performed according to the literature [1], with some modifications. FRAP solution consisted of a mixture of 1 mL of TPTZ, 1 mL of iron (III) chloride and 10 mL of acetate buffer (300 mmol/L, pH 3.6), placed in the oven at 37 ºC for 10 minutes; 10 mL of this mixture were diluted in 20 mL of acetate buffer. In 96-well plates, 270 µL of FRAP solution and 30 µL of aqueous solution of (pyrano)anthocyanins extract were mixed and the absorbance at 593 nm at 37 ºC was measured at 0 and 4 minutes on a plate reader (Biotek Powerwave XS). Results were expressed as Trolox equivalents. DPPH assay was performed according to the literature [1], with some modifications. In 96-well plates, 270 µL of DPPH solution (prepared in methanol at a concentration 24.2 µg/mL) were mixed with 30 µL of sample and the absorbance at 515 nm was recorded every 5 minutes, for 20 minutes, on a plate reader (Biotek Powerwave XS). Results were expressed as Trolox equivalents. 5 replicates were made for each real triplicate.

Supplementary Results

- **Characterization of anthocyanin and pyranoanthocyanin extracts**

a)

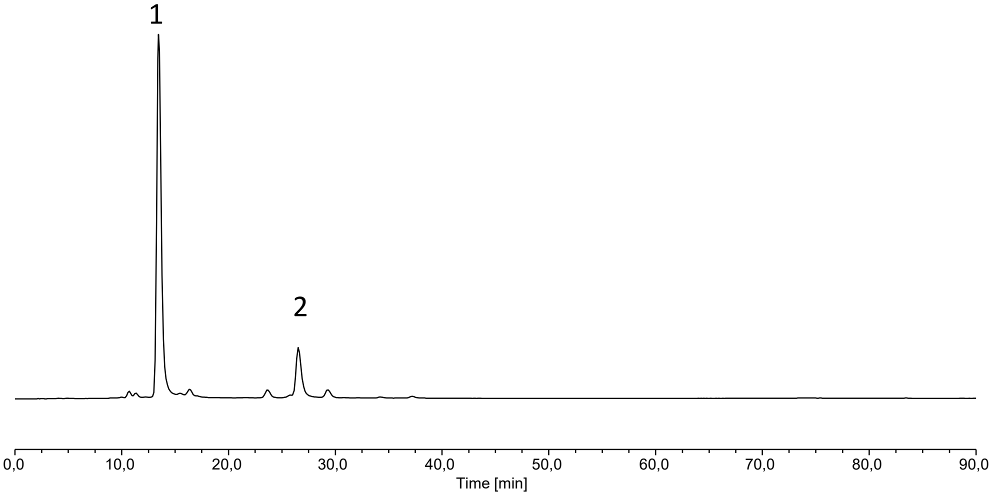

b)

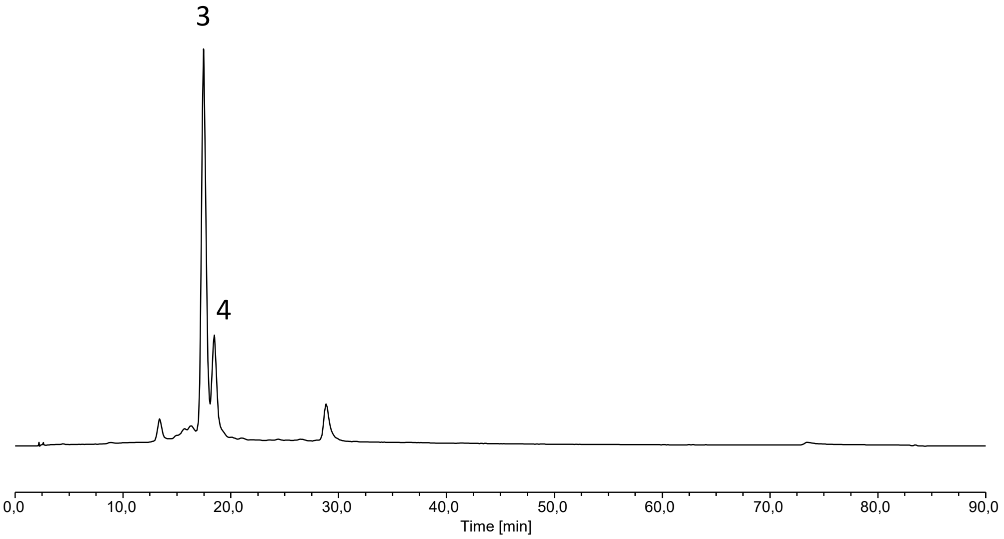

c)

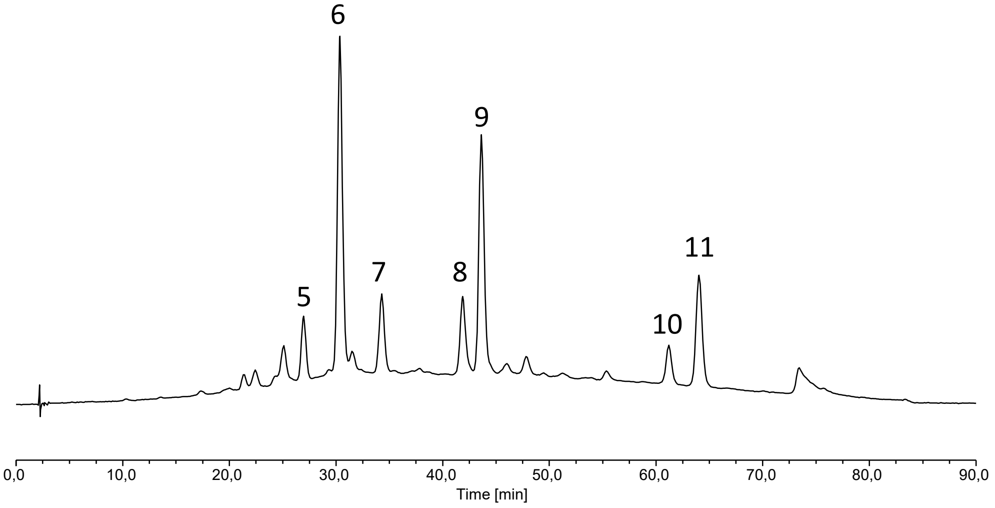

-

Figure S1. Chromatographic profile of a) blackberry anthocyanins, b) elderberry and c) red wine pyranoanthocyanins, at 520 nm. 1: Cyanidin-3-O-glucoside; 2: Cyanidin; 3: Carboxypyranocyanidin-3-glucoside; 4: Carboxypyranocyanidin-3-sambubioside; 5: Carboxypyranopeonidin-3-glucoside , 6: Carboxypyranomalvidin-3-glucoside ; 7: Carboxypyranomalvidin-3-(6′′-acetylglucoside); 8: Carboxypyranopeonidin-3-(6′′-coumaroylglucoside); 9: Carboxypyranomalvidin-3-(6′′-coumaroylglucoside); 10: Peonidin-3-(6′′-coumaroylglucoside); 11: Malvidin-3-(6′′-coumaroylglucoside)

Table S1. Chemical composition and antioxidant activity of the anthocyanin and pyranoanthocyanin extracts.

Identification	Extract blackberry anthocyanins (mg ± SD)	Extract elderberry Pyranoanthocyanins (mg ± SD)	Extract Red wine Pyranoanthocyanins (mg ± SD)
Anthocyanins/pyranoanthocyanins	0.62 ± 0.07	0.610 ± 0.008	0.18 ± 0.002
Low weight polyphenols	0.0024 ± 0.0004	0.00073 ± 0.00005	0.010 ± 0.002
Flavonols	0.006 ± 0.002	0.03 ± 0.01	0.056 ± 0.006
Protein content	0.0519 ± 0.0019	0.0463 ± 0.0069	0.096 ± 0.002
Total lipids	0.10 ± 0.02	0.12 ± 0.05	0.05 ± 0.01
Sugar content	0.0258 ± 0.0041	0.0345 ± 0.0051	0.0184 ± 0.0023
Trolox equivalents			
Antioxidant activity (µmol ± SD)	66 ± 5	63 ± 8	52 ± 4
Antiradicalar activity (µmol ± SD)	52 ± 16	61 ± 12	63 ± 12

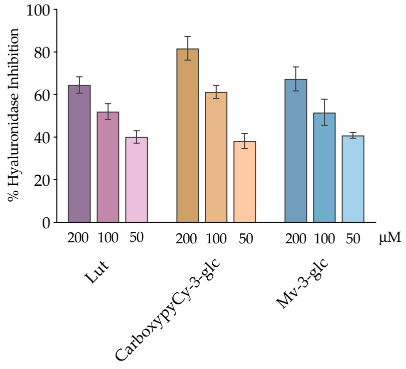

Figure S2. Hyaluronidase inhibition rates in the presence of increasing concentrations of Lut, CarboxypyCy-3-glc and Mv-3-glc.

- **Molecular docking studies (Hyaluronidase)**

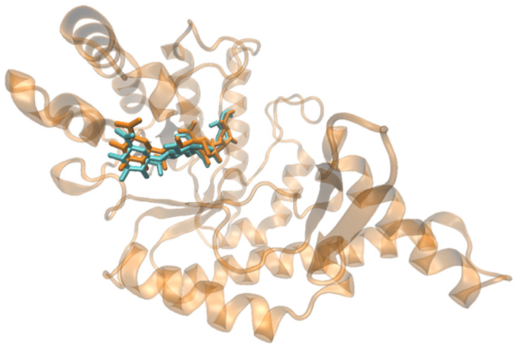

Figure S3 – Superimposition of the docking and crystallographic geometries of the tetrasaccharide bound into the active site of the hyaluronidase. Enzyme is depicted in cartoon and colored in orange, while the tetrasaccharide is represented with sticks and colored in blue (docking pose) or orange (X-ray pose). Hydrogen atoms are not represented to simplify the visualization.

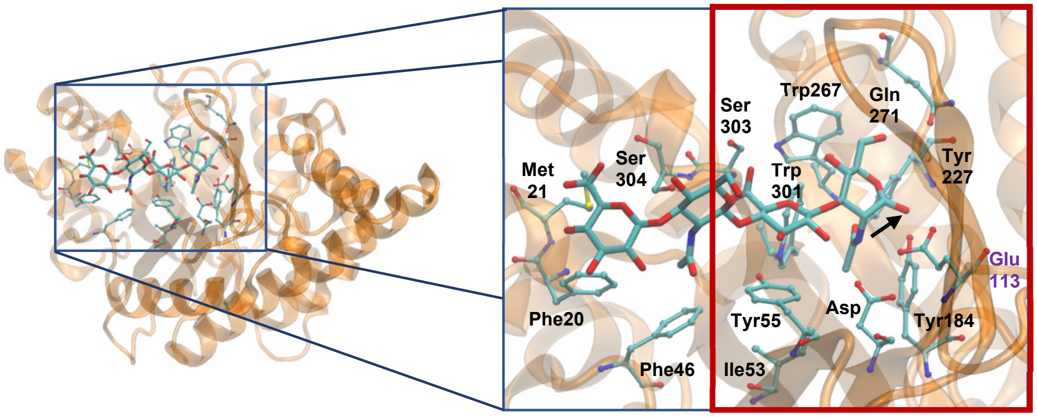

Figure S4 – Representation of the structure of hyaluronidase complexed with tetrasaccharide, showing the interacting residues of the active site. The arrow is pointing to the atom where the cleavage occurs. The enzyme is depicted in cartoon and colored in orange, the tetrasaccharide is represented with sticks and colored by atom type, whilst the interacting residues are depicted in ball-and-sticks and colored by atom type. Hydrogen atoms are not represented to simplify the visualization.

Table S2. Values of ΔGbinding for the best molecular docking solutions obtained for each compound. The closest group to Glu113 was also identified as well as the respective distance between oxygen atoms. The interacting residues of the active site were identified.

Compound	$\Delta G_{binding}$ / kcal/mol	Nearest group to Glu113	Smallest distance to Glu113 / Å	Interacting residues of the active site (distance < 4 Å)
Tetrasaccharide	-3.55	O-C4 - glucose	2.79	Ile53, Tyr55, Asp111, Glu113, Tyr184, Tyr227, Trp267, Gln271, Trp301, Ser303 and Ser304
Que	-5.94	ring B	2.94	Tyr55, Asp111, Glu113, Tyr184, Tyr227, Trp301, Ser303, Ser304 and Asp305
Que-3-glc	-5.83	glucose	3.90	Tyr55, Asp56, Asp111, Glu113, Tyr184, Tyr227, Trp301, Ser303, Ser304 and Asp305
	-5.58	ring B	3.00	Tyr55, Asp56, Asp111, Phe112, Glu113, Ser114, Tyr184, Trp301, Ser303 and Ser304
	-4.17	rings A and C	3.42	Tyr55, Asp111, Glu113, Tyr184, Tyr227, Gln271, Arg274, Trp301, Ser303, Ser304 and Asp305
Cy-3-glc	-6.34	ring B	2.73	Tyr55, Asp56, Asp111, Phe112, Glu113, Ser114, Tyr184, Tyr227, Trp301 and Ser304
	-5.97	glucose	2.64	Tyr55, Asp56, Asp111, Glu113, Tyr184, Tyr227, Trp301, Ser303 and Ser304
Cy-3-glc (chalcone)	-5.03	ring B	4.21	Tyr55, Asp56, Asp111, Phe112, Glu113, Tyr184, Tyr227, Arg274, Trp301, Ser303 and Ser304
Lut	-6.31	ring B	3.36	Tyr55, Asp111, Glu113, Tyr184, Tyr227, Trp301, Ser303 and Ser304
Lut (chalcone)	-6.01	ring B	4.15	Tyr55, Asp56, Asp111, Phe112, Glu113, Ser114, Tyr184, Tyr227 and Trp301
CarboxypyCy-3-glc	-8.06	glucose	2.61	Tyr55, Asp56, Asp111, Phe112, Glu113, Tyr184, Tyr227, Arg274, Trp301, Ser303, Ser304 and Asp305
	-6.06	ring B	4.59	Tyr55, Asp56, Asp111, Glu113, Tyr184, Tyr227, Trp267, Gln271, Trp301, Ser303, Ser304 and Asp305
MethylpyCy-3-glc	-7.15	glucose	2.60	Tyr55, Asp56, Asp111, Phe112, Glu113, Tyr184, Tyr227, Arg274, Trp301, Ser303 and Ser304
Mv-3-glc	-5.80	ring B	4.55	Pro18, Ile53, Tyr55, Asp56, Asp111, Glu113, Tyr184, Tyr227, Trp301 and Ser303
	-5.79	glucose	2.74	Ile53, Tyr55, Asp56, Asp111, Glu113, Tyr184, Tyr227, Trp301, Gly302, Ser303, Ser304 and Asp305
	-5.76	rings A and C	2.41	Tyr55, Asp56, Asp111, Phe112, Glu113, Ser114, Tyr184, Tyr227 and Trp301
Mv-3-glc (chalcone)	-6.00	rings A and C	2.72	Tyr55, Asp111, Phe112, Glu113, Ser114, Tyr184, Tyr227, Trp301, Ser303 and Ser304
	-5.28	glucose	2.93	Ile53, Tyr55, Asp56, Asp111, Glu113, Ser114, Tyr184, Tyr227 and Trp301
DeoxyMv	-6.45	ring B	3.89	Pro18, Ile53, Tyr55, Asp111, Glu113, Tyr184, Tyr227, Trp301, Gly302, Ser303 and Ser304
DeoxyMv (chalcone)	-5.27	ring B	4.26	Ile53, Tyr55, Asp111, Glu113, Tyr184, Tyr227, Trp301, Gly302, Ser303 and Ser304
CarboxypyMv-3-glc (40%)	-6.42	ring B	4.94	Ile53, Tyr55, Asp56, Asp111, Tyr184, Tyr227, Trp301, Ser303, Ser304 and Asp305

CarboxypyMv-3-glc (60%)	-6.15	ring B	4.78	Tyr55, Asp56, Asp111, Glu113, Tyr184, Tyr227, Trp301, Gly302, Ser303, Ser304 and Asp305
MethylpyMv-3-glc	-6.77	ring B	4.59	Tyr55, Asp56, Asp111, Glu113, Tyr184, Tyr227, Trp301, Gly302, Ser303, Ser304 and Asp305

References

1. Azevedo, J.; Fernandes, I.; Lopes, P.; Roseira, I.; Cabral, M.; Mateus, N.; Freitas, V. Migration of Phenolic Compounds from Different Cork Stoppers to Wine Model Solutions: Antioxidant and Biological Relevance. *Eur. Food Res. Technol.* **2014**, *239*, 951-960, doi:10.1007/s00217-014-2292-y.
2. Soares, S.; Soares, S.; Brandão, E.; Guerreiro, C.; Mateus, N.; de Freitas, V. Oral interactions between a green tea flavanol extract and red wine anthocyanin extract using a new cell-based model: insights on the effect of different oral epithelia. *Sci Rep* **2020**, *10*, 12638, doi:10.1038/s41598-020-69531-9.
3. Teixeira, N.; Mateus, N.; Freitas, V.; Oliveira, J. Wine Industry By-product: Full Polyphenolic Characterization of Grape Stalks. *Food Chem.* **2018**, *268*, 110-117, doi:10.1016/j.foodchem.2018.06.070.
4. Fernandes, I.; Faria, A.; Azevedo, J.; Soares, S.; Calhau, C.; Freitas, V.; Mateus, N. Influence of Anthocyanins, Derivative Pigments and Other Catechol and Pyrogallol-Type Phenolics on Breast Cancer Cell Proliferation. *J. Agric. Food Chem.* **2010**, *58*, 3785-3792, doi:10.1021/jf903714z.
5. Bradford, M.M. A Rapid and Sensitive Method for the Quantitation of Microgram Quantities of Protein Utilizing the Principle of Protein-dye Binding. *Anal. Biochem.* **1976**, *72*, 248-254, doi:10.1016/0003-2697(76)90527-3.
6. Podolec, P.; Szabó, A.H.; Blaško, J.; Kubinec, R.; Górová, R.; Višňovský, J.; Gnipová, A.; Horváth, A.; Bierhanzl, V.; Hložek, T., et al. Direct silylation of Trypanosoma brucei metabolites in aqueous samples and their GC-MS/MS analysis. *J Chromatogr B Analyt Technol Biomed Life Sci* **2014**, *967*, 134-138, doi:10.1016/j.jchromb.2014.07.023.
